# An RFX transcription factor regulates ciliogenesis in the closest living relatives of animals

**DOI:** 10.1016/j.cub.2023.07.022

**Published:** 2023-08-07

**Authors:** Maxwell C. Coyle, Adia M. Tajima, Fredrick Leon, Semil P. Choksi, Ally Yang, Sarah Espinoza, Timothy R. Hughes, Jeremy F. Reiter, David S. Booth, Nicole King

**Affiliations:** 1 Howard Hughes Medical Institute and Department of Molecular and Cell Biology, University of California, Berkeley, Berkeley, CA, 94720; 2 Department of Biochemistry and Biophysics, University of California, San Francisco, CA, 94158; 3 Cardiovascular Research Institute, University of California, San Francisco, CA, 94158; 4 Donnelly Centre for Cellular and Biomolecular Research, Toronto, Canada, M5S 3E1; 5 Department of Molecular Genetics, University of Toronto, Canada, M5S 3E1; 6 Chan Zuckerberg Biohub, San Francisco, CA, 94158

**Keywords:** cilia, ciliogenesis, transcription factor, RFX, FoxJ1, evolution, choanoflagellate, animal origins

## Abstract

Cilia allowed our protistan ancestors to sense and explore their environment, avoid predation, and capture bacterial prey^1–3^. Regulated ciliogenesis was likely critical for early animal evolution^2,4–6^ and, in modern animals, deploying cilia in the right cells at the right time is crucial for development and physiology. Two transcription factors, RFX and FoxJ1, coordinate ciliogenesis in animals^7–9^ but are absent from the genomes of many other ciliated eukaryotes, raising the question of how the regulation of ciliogenesis in animals evolved^10,11^. By comparing the genomes of animals with those of their closest living relatives, the choanoflagellates, we found that the genome of their last common ancestor encoded at least three RFX paralogs and a FoxJ1 homolog. Disruption of the RFX homolog *cRFXa* in the model choanoflagellate *Salpingoeca rosetta* resulted in delayed cell proliferation and aberrant ciliogenesis, marked by the collapse and resorption of nascent cilia. In *cRFXa* mutants, ciliogenesis genes and *foxJ1* were significantly down-regulated. Moreover, the promoters of *S. rosetta* ciliary genes are enriched for DNA motifs matching those bound by the cRFXa protein *in vitro*. These findings suggest that an ancestral *cRFXa* homolog coordinated ciliogenesis in the progenitors of animals and choanoflagellates and that the selective deployment of the RFX regulatory module may have been necessary to differentiate ciliated from non-ciliated cell types during early animal evolution.

## eTOC Blurb

The regulation of ciliogenesis was critical for early animal evolution but has not been studied in close animal relatives. Coyle *et al*. show the ciliogenesis regulatory function of a choanoflagellate RFX gene, suggesting that the co-option of ciliary genes under novel transcriptional regulators preceded the evolution of animal development.

## Results and Discussion

### Choanoflagellates express orthologs of animal cilia-associated transcription factors

Key features of the progenitors of animals can be inferred by comparing animals with their closest living relatives, the choanoflagellates^6,12,13^. Choanoflagellate cells feature a distinctive “collar complex” composed of a single apical cilium surrounded by a collar of actin-filled microvilli^12,13^. Structural conservation of cilia across eukaryotic diversity suggests that the last common ancestor of eukaryotes had a cilium^1,14^ and that cilia of choanoflagellates and animals are homologous^15^.

**Figure 1.**
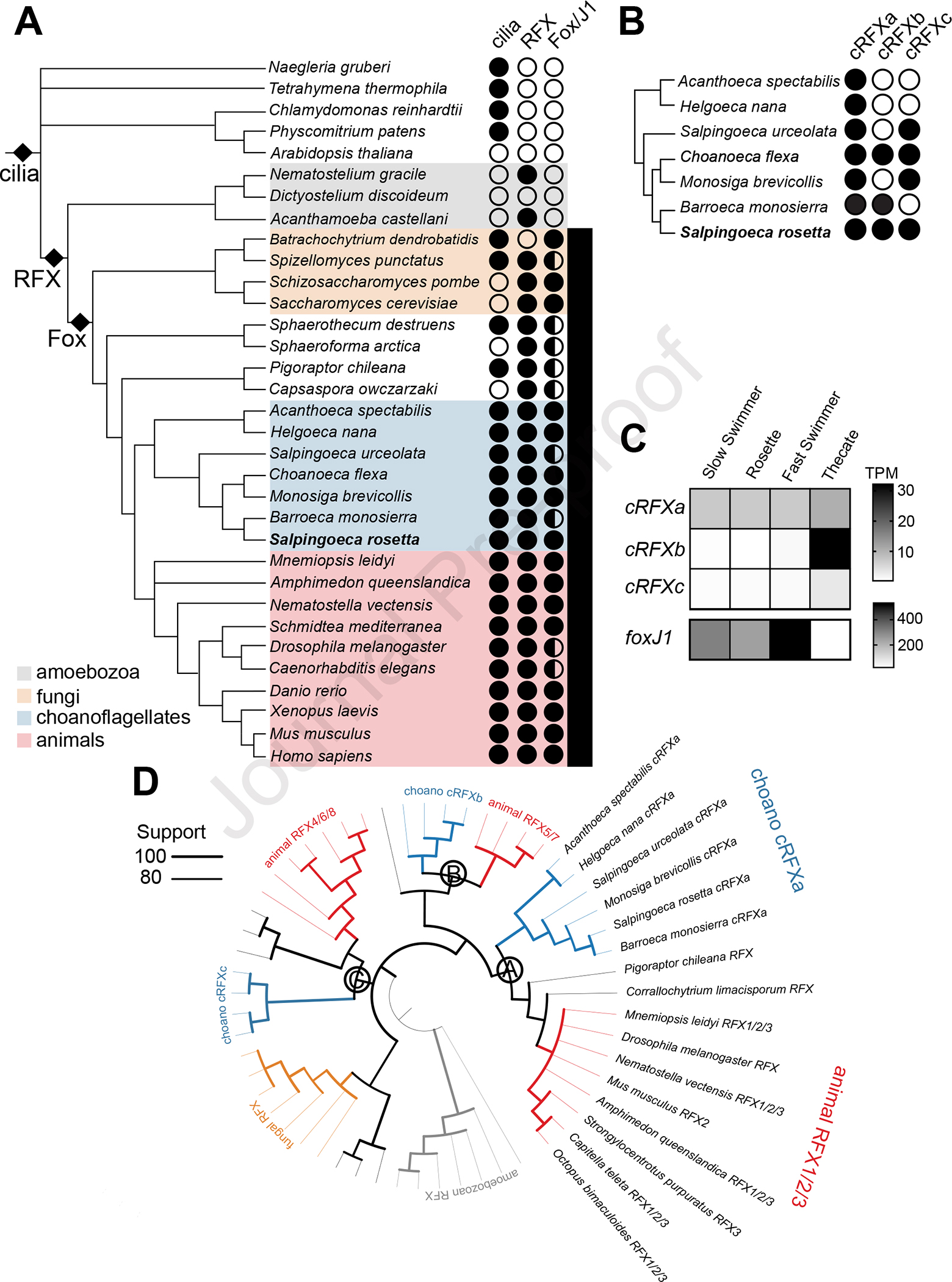
The evolutionary history of cilia-associated transcription factors and their expression in choanoflagellates. (A) Cilia evolved before the emergence of RFX and Fox TFs. The presence (filled circle) or absence (open circle) of RFX and Fox domain proteins is indicated for diverse eukaryotes (Files S1, S2; STAR Methods). Half shading in the Fox/J1 column indicates the presence of Fox family members, while full shading indicates the presence of a putative FoxJ1 homolog reciprocal best BLAST hit with either the *Xenopus laevis* or *Schmidtea mediterranea* FoxJ1 (File S2; STAR Methods). Cilia have been observed in most eukaryotic lineages, indicating a cilium was present in the last eukaryotic common ancestor. RFX TFs are more phylogenetically restricted, having been found across opisthokonts and amoebozoans, while Fox TFs are nearly entirely restricted to opisthokonts (see STAR Methods for rare exceptions to these patterns.) All choanoflagellates express Fox TF homologs and FoxJ1 orthologs were detected in most choanoflagellate species. Species tree represents a consensus of recent well-supported eukaryotic and clade-specific phylogenies^69–73^. (B) The *cRFXa* sub-family is widespread in choanoflagellates. RFX family relationships were determined using maximum-likelihood phylogenetic trees built by IQ-TREE^74^ (Figure S1A; File S1). All RFX TFs in choanoflagellates grouped into one of three well-supported sub-families: *cRFXa*, *cRFXb*, and *cRFXc.* For representative choanoflagellates, the presence (filled circle) or absence (open circle) of each sub-family is indicated. While *cRFXa* was detected in all cultured choanoflagellates that have been sequenced, *cRFXb* and *cRFXc* were restricted to subsets of choanoflagellate diversity. (C) *cRFXa* is expressed in all surveyed *S. rosetta* life history stages. *S. rosetta* can transition between multiple colonial and solitary cell types^30^, including slow swimmers, rosettes, fast swimmers, and thecate cells. Cells in all life history stages depicted here bear motile cilia. RNA-seq analysis showed that only *cRFXa* is expressed above background levels (average TPM [transcripts per million] ≥ 1) in all cell types. *cRFXb* and *cRFXc* are only expressed above background levels in thecate cells (File S3). *foxJ1* is expressed in all cell types and most highly in fast swimmers (File S3). Shading indicates average TPM value of the gene across three biological replicates. Note the separate scale bars for *RFX* and *foxJ1* expression levels due to the approximately ten-fold difference in maximum expression level between these genes. (D) Choanoflagellate *cRFXa* genes form a clade with the animal *RFX1/2/3* family. This maximum-likelihood phylogenetic tree includes RFX sequences from diverse opisthokonts and amoebozoans (File S1). Width of branches indicates scales with UFboot support for the ancestral node and all nodes with less than 75% bootstrap support are collapsed. Labels A, B, and C indicate ancestral nodes of homologous choanoflagellate/animal RFX sub-families. Node A has 81% bootstrap support, Node B has 81% bootstrap support, and Node C has 85% bootstrap support. Branch lengths do not scale with evolutionary distance in this rendering. See Figure S1B for full annotated version of this phylogeny, including branch lengths, bootstrap values, and all species names. See Figure S1C for phylogenetic trees built with different trimming and reconstruction algorithms. See also Data S1.

RFX and FoxJ1 are two transcription factors (TFs) that regulate animal ciliogenesis. Loss of either RFX or FoxJ1 function in animals reduces the transcription of many ciliary genes^16–18^ and results in ciliogenesis defects^8,9,19–23^. Despite their essentiality for proper ciliogenesis in animals, RFX and FoxJ1 are either missing (e.g., in *Chlamydomonas, Naegleria*, and ciliates), of unknown function (e.g., in choanoflagellates and chytrids), or of non-ciliary function (e.g., in ascomycete fungi^24–27^) in non-animals (Figure 1A). To better understand the phylogenetic distribution of *RFX* and *foxJ1* genes, we used DNA-binding domain (DBD) sequences from diverse FoxJ1 and RFX protein sequences to query EukProt^28^ (Figure 1A; STAR Methods; Files S1, S2). Confirming previous reports^6,29^, we found an ortholog of animal *foxJ1* genes in *S. rosetta*. Choanoflagellate RFX genes fall into three paralogous sub-families, provisionally named *cRFXa*, *cRFXb*, and *cRFXc* (Figure 1B; Figure S1A). *cRFXa* homologs were detected in nearly all choanoflagellate species analyzed, while *cRFXb* and *cRFXc* homologs have more restricted phylogenetic distributions (Figure 1B).

The life history of *S. rosetta* includes transitions between diverse ciliated cell types – including slow swimmers, fast swimmers, thecate cells, and multicellular rosettes^30^. We found that *cRFXa* was transcribed in each life history stage, while *cRFXb* and *cRFXc* expression was restricted to thecate cells (Figure 1C; File S3). *foxJ1* was down-regulated in thecate cells and up-regulated in fast swimmers, a starvation-induced cell type with longer cilia and a faster swimming velocity^31^ (Figure 1C; File S3).

Phylogenetic analysis of RFX protein sequences from diverse opisthokonts and amoebozoans recovered the three choanoflagellate sub-families (cRFXa, cRFXb, and cRFXc), three RFX sub-families previously reported in animals (RFX1/2/3, RFX4/6/8 and RFX5/7^11^), and distinct clades of amoebozoan and fungal RFX proteins (Figure 1D; Figure S1B). The cRFXa sub-family branched with the animal RFX1/2/3 sub-family, which regulates ciliogenesis in many tissues across diverse animals ^7,8,19,21^ (Figure 1D; Figure S1B, S1C). The cRFXb and cRFXc sub-families grouped with the animal RFX5/7 and RFX4/6/8 sub-families, respectively, both of which serve diverse functions in animals and regulate ciliogenesis only in specific contexts^32–35^. We thus infer that the last common ancestor of choanoflagellates and animals encoded at least three *RFX* paralogs, one related to modern-day *RFX1/2/3/cRFXa* genes, one related to *RFX5/7/cRFXb* genes, and one related to *RFX4/6/8/cRFXc* genes (Figure 1D).

### *Disruption of S. rosetta* cRFXa *delays cell proliferation and ciliogenesis*

**Figure 2.**
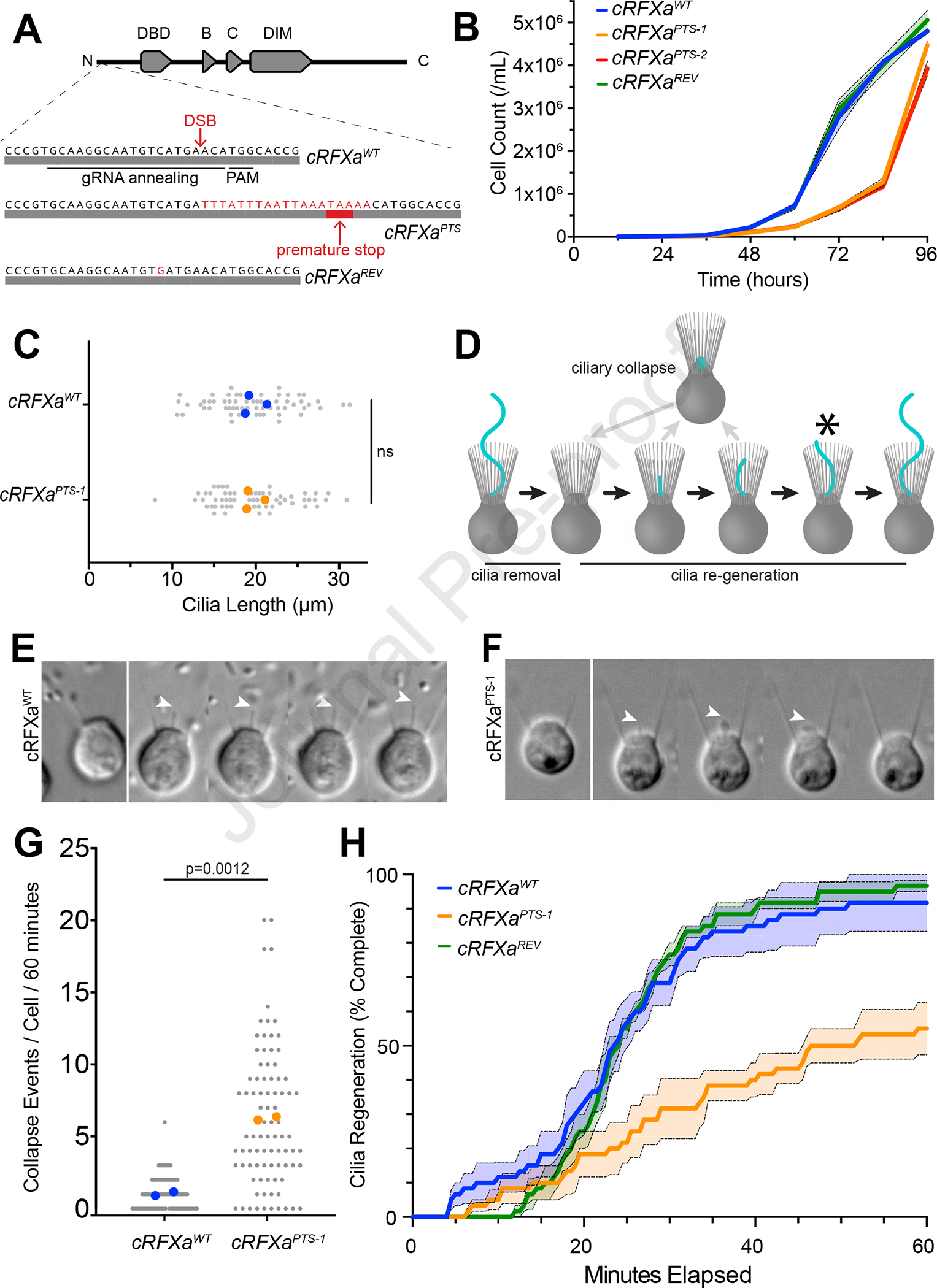
Truncation of cRFXa results in cell proliferation and ciliogenesis defects. (A) The *S. rosetta cRFXa* locus encodes a protein that contains an N-terminal DNA-binding domain (DBD) followed by two conserved domains of unknown function (B, C) and a dimerization domain (DIM)^7^. The *cRFXa* locus was targeted by a guide RNA (gRNA) that anneals to an exon near the 5’ end of the gene coupled with a homology-directed repair template that inserts a cassette (TTTATTAATTAAATAAA) that encodes an early stop codon (* in translation product, grey shaded letters). The edited allele is called *cRFXa*^*PTS*^ (for Premature Termination Signal^36^) and codes for a truncated polypeptide of 24 amino acids. Two independent *cRFXa*^*PTS*^ mutants, *cRFXa*^*PTS–1*^ and *cRFXa*^*PTS–2*^, were recovered. The *cRFXa*^*PTS–1*^ strain was reverted to a wild-type polypeptide sequence to create the *cRFXa*^*REV*^ strain, which harbors a synonymous GTC→GTG (Valine) that allows its genotype to be distinguished from that of *cRFXa*^*WT*^ cells. DSB = double-strand break, PAM = protospacer adjacent motif. Numbers indicate amino acid positions in coding DNA sequence. See Figure S2A for genotyping confirmation. (B) Truncation of cRFXa in the *cRFXa*^*PTS–1*^ and *cRFXa*^*PTS–2*^ strains resulted in delayed cell proliferation compared to *cRFXa*^*WT*^ and *cRFXa*^*REV*^ cells. Cells were diluted to 1,000 cells/ml and triplicate samples were collected and counted every 12 hours for 96 hours. The mean values were plotted with the standard error of the mean shown as dotted lines. See Figures S2B and S2C for growth curves of other TF mutant strains. (C) Cilia lengths were comparable in *cRFXa*^*W*T^ (19.73 μm) and *cRFXa*^*PTS–1*^ (19.63 μm) cells. Cilia lengths in randomly selected cells from three biological replicates were analyzed (see Materials and Methods), measuring 20 cells/genotype/replicate, for 60 cells total/replicate. Colored dots show replicate mean values and grey dots show the lengths of individual cilia. Unpaired t-test compares mean values of biological replicates (n = 3), p-value = 0.959. ns = not significant. (D) Choanoflagellate ciliogenesis can be synchronized and quantified following ciliary removal. To this end, *S. rosetta* cells were treated with 10% glycerol and cold-shocked (STAR Methods), which results in the severing of cilia. We observed that nascent cilia sometimes collapse and resorb before a new round of ciliary growth begins (grey arrows). The point at which the growing cilium passed the edge of the microvillar collar was used as a marker of successful ciliogenesis (asterisk). (E) A representative time series shows a *cRFXa*^*WT*^ cell in the process of ciliogenesis, from cilia removal (00:00 mm:ss) to growth (15:30–17:00 mm:ss). The nascent cilium (arrowhead) extended as a thin, straight protrusion; ciliary beating had not begun yet. The cell shifted slightly in position under the coverslip between 00:00 and 15:30. Scale bar = 5 μm. See Video S1 and S2 for complete examples of *cRFXa*^*WT*^ regeneration. (F) A representative time series shows a *cRFXa*^*PTS–1*^ cell in the process of ciliogenesis. Arrowhead marks a nascent cilium that collapsed (20:00 time point) and resorbed back into the cell. Resorption here was complete in one minute, which was typical. The cell shifted slightly in position under the coverslip between 00:00 and 19:30. Scale bar = 5 μm. See Video S3 and S4 for complete examples of *cRFXa*^*WT*^ regeneration. (G) Nascent cilia in *cRFXa*^*PTS–1*^ cells collapse more frequently than *cRFXa*^*WT*^ cells during ciliogenesis. For each of two biological replicates, 20+ randomly selected cells were scored for the number of ciliary collapses during a 60-minute ciliary regeneration period. Colored dots show mean values of each biological replicate and grey dots show values for individual cells. The mean number of collapses (across biological replicates) was 1.00 collapses/cell/60 minutes for *cRFXa*^*WT*^ and 6.24 for *cRFXa*^*PTS-1*^. Unpaired t-test compares mean values of biological replicates (n = 2), p-value = 0.0012. (H) *cRFXa*^*PTS–1*^ cells are delayed in ciliary regeneration relative to *cRFXa*^*WT*^ and *cRFXa*^*REV*^ cells. Graph shows the percent of cells that have completed ciliary regeneration as a function of time (three biological replicates, 20 cells each). Regeneration was defined as the point at which the cilium grows past the collar (see panel D). Dotted lines show standard error of the mean across three replicates. See Figure S2D–F for ciliary regeneration curves for other TF mutant strains. See also Data S1.

To investigate the function of the *cRFXa*, *cRFXb*, *cRFXc*, and *foxJ1* genes in *S. rosetta*, we used CRISPR-mediated gene editing^36^ to introduce an early stop codon near the 5’ ends of each gene (Figure 2A; Figure S2A; File S4). The resulting strains were cultured under conditions that favor the proliferation of slow swimmers, the cell type used for all experiments here (STAR Methods). Mutants for *foxJ1*, *cRFXb*, or *cRFXc* showed normal growth and displayed no obvious phenotypic defects (Figure S2B, C). In contrast, two independently isolated *cRFXa* mutant lines, each encoding a truncated allele of *cRFXa* (*cRFXa*^*PTS–1*^ and *cRFXa*^*PTS–2*^), proliferated more slowly than a wild-type control (*cRFXa*^*WT*^; Figure 2B). A strain in which the *cRFXa*^*PTS–1*^ allele was reverted to the wild-type amino acid sequence (*cRFX*^*REV*^) had comparable growth to that of *cRFXa*^*WT*^ cells, confirming that the growth defect in the *cRFXa*^*PTS–1*^ strain was a direct result of the cRFXa truncation (Figure 2B).

Cilia lengths were indistinguishable between *cRFXa*^*WT*^ and *cRFXa*^*PTS–1*^ cells (Figure 2C), but this did not reveal the dynamics of ciliogenesis itself. Therefore, we performed live imaging of ciliary regeneration^37^ (Figure 2D; STAR Methods). In *cRFXa*^*WT*^ cells, the nascent cilium emerged rapidly and proceeded to lengthen (Figure 2E; Video S1, S2). In comparison, the nascent cilia of *cRFXa*^*PTS–1*^ mutant cells collapsed and were resorbed into the cell frequently (6.24 ciliary collapse events/cell/60 minutes compared to 1.00 for *cRFXa*^*WT*^ cells; p-value = 0.0012, unpaired t-test; Figures 2F, G; Videos S3, S4).

To quantify the rate of ciliogenesis, we established a metric by which cells were scored as having a regenerated cilium once the apical tip of the cilium grew past the apical boundary of the microvillar collar (Figure 2D). Within 60 minutes after ciliary removal, only 55% of *cRFXa*^*PTS–1*^ mutant cells and 50% of *cRFXa*^*PTS–2*^ cells had successfully regenerated their cilium, whereas 90% of *cRFXa*^*WT*^ cells and 97% of *cRFXa*^*REV*^ cells completed ciliary regeneration (Figure 2H; Figure S2D). In contrast, the *cRFXb*^*PTS*^, *cRFXc*^*PTS*^, and *foxJ1*^*PTS*^ mutants did not display any detectable ciliogenesis defect (Figure S2E, F, G). Moreover, a *cRFXa*^*PTS–1*^*;foxJ1*^*PTS*^ double mutant, generated by CRISPR editing of *foxJ1* in the *cRFXa*^*PTS–1*^ background, showed no additional defect in ciliary regeneration beyond that observed in *cRFXa*^*PTS–1*^ cells (Figure S2H). In summary, *cRFXa* is required for proper cilia regeneration in *S. rosetta* slow swimmers, while *cRFXb*, *cRFXc, and foxJ1* are not.

### cRFXa *promotes transcription of conserved ciliogenesis genes and foxJ1*

**Figure 3.**
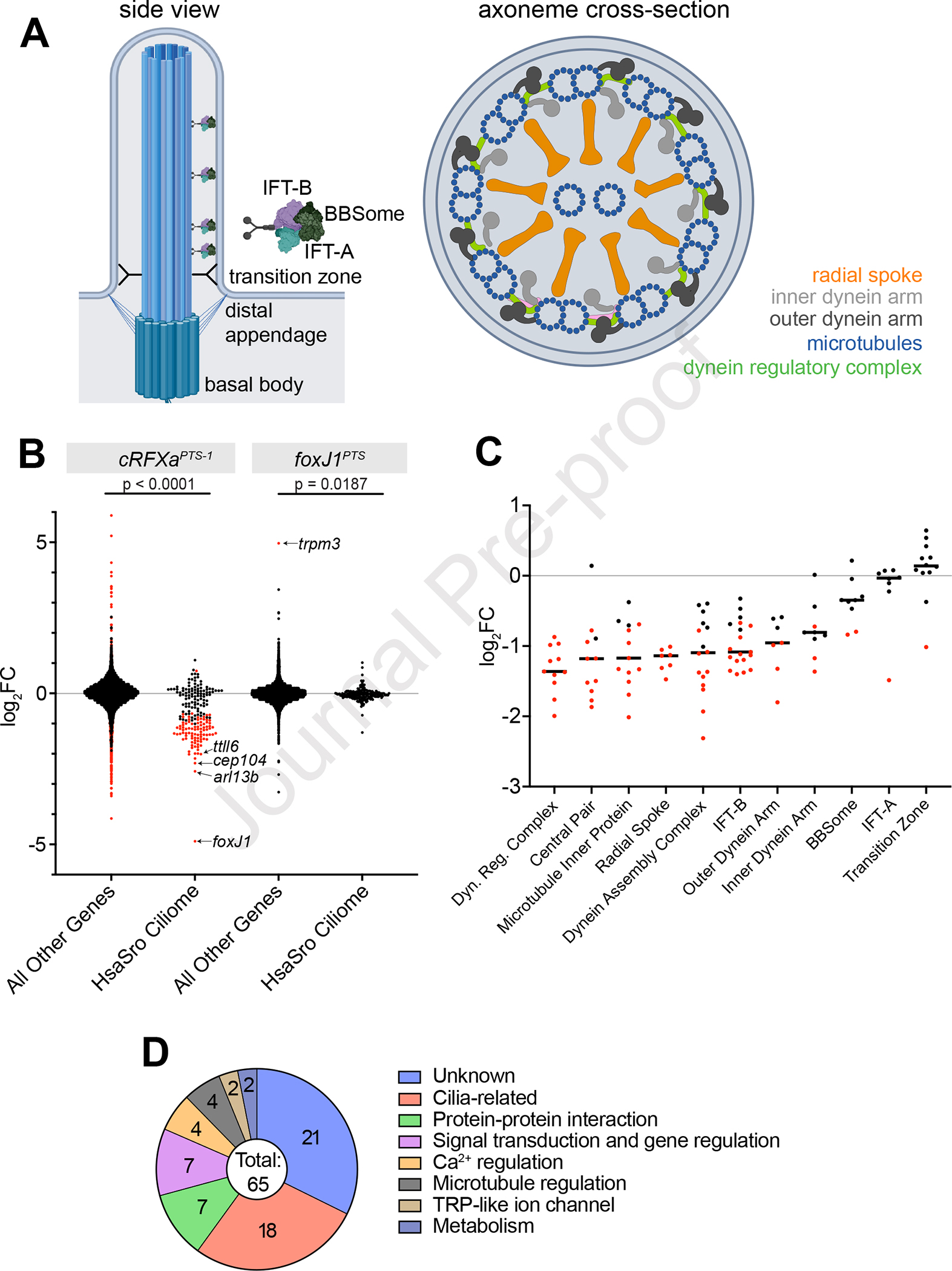
*cRFXa*^*PTS*^ cells down-regulate conserved ciliary genes. (A) Eukaryotic motile cilia are constructed from conserved macromolecular complexes encoded by dozens of genes (File S6). The side view of a cilium shows how the basal body, which nucleates the microtubules of the cilium, docks to the cell membrane. Intraflagellar transport (IFT) trains traverse in both anterograde and retrograde directions to shuttle ciliary components to the growing tip. Axoneme cross-section shows the organization of microtubule doublets in the cilium as well as the inter-doublet links and dynein arms that power ciliary motility. (B) Ciliary genes, including *foxJ1*, were significantly down-regulated in the *cRFXa*^*PTS–1*^ mutant compared to *cRFXa*^*WT*^ cells. Shown are log_2_FC values for HsaSro conserved ciliary genes (n = 201), compared to all other predicted genes in the *S. rosetta* genome, for both *cRFXa*^*PTS–1*^ and *foxJ1*^*PTS*^ strains, relative to wild-type cells. All strains were sequenced while cells were in mid-log growth phase as slow swimmers. Red dots indicate genes whose differential expression was called as significant by edgeR using a false discovery rate (FDR) cut-off of < 0.001. For *cRFXa*^*PTS–1*^, the average log_2_fold-change of all ciliary genes was −0.68 compared to −0.017 for non-ciliary genes (Mann-Whitney p-value < 0.0001). For *foxj1*^*PTS*^, the average log_2_fold-change of all ciliary genes was −0.04 compared to −0.0086 for non-ciliary genes (Mann-Whitney p-value = 0.0187). See Figure S3 for RNA-seq expression of *S. rosetta* ciliary genes identified by mass spectrometry^43^. (C) Many categories of ciliary genes were down-regulated in *cRFXa*^*PTS–1*^ cells. For each category, the horizontal bar shows the average log_2_FC value for genes in that category, while dots indicate the expression changes of individual genes. Red dots indicate a gene with an edgeR false discovery rate (FDR) < 0.001. (D) Predicted functions for all 65 genes down-regulated more than four-fold (log_2_FC < −2) in the *cRFXa*^*PTS–1*^ mutant. Categories were called based on protein domain annotation by InterProScan and the closest human BLAST hit (File S6). See also Data S1.

To investigate how disruption of *cRFXa* in *S. rosetta* leads to aberrant ciliogenesis, we next investigated the transcriptional profiles of *cRFXa*^*WT*^ and *cRFXa*^*PTS–1*^ cells. In animals, the ciliary phenotypes of RFX loss-of-function mutants are associated with reduced expression of many ciliary genes^17,18,38,39^ and we hypothesized that the same might be true for choanoflagellates. To identify candidate ciliary genes in *S. rosetta*, we curated the “HsaSro conserved ciliome,” a list of 201 genes that (1) are required for proper assembly of cilia in humans, (2) have a well-characterized molecular function, and (3) are conserved between humans and *S. rosetta* (STAR Methods; File S6). The HsaSro conserved ciliome includes axonemal dyneins, genes involved in intraflagellar transport (IFT), radial spokes, the BBSome, tubulin modifiers, the ciliary transition zone, ciliary vesicle formation, and more (Figure 3A).

Of the 201 genes in the HsaSro conserved ciliome, 93 were significantly down-regulated in *cRFXa*^*PTS–1*^ cells compared to *cRFXa*^*WT*^ cells (edgeR FDR < 0.001; Figure 3B; Files S5, S6). The down-regulated ciliary genes had slightly more than a 2-fold reduction in expression (Figure 3B, C), while genes not in the HsaSro conserved ciliome had, on average, no change in expression (Figure 3B). Among the most down-regulated ciliary genes in *cRFXa*^*PTS–1*^ cells were the ciliary GTPase *arl13B*^40^, the ciliary tip component *cep104*^41^, and the tubulin glutamylation enzyme *ttll6*^42^ (Figure 3B). Moreover, genes previously detected in the *S. rosetta* ciliome by mass spectrometry^43^ were preferentially down-regulated in *cRFXa*^*PTS–1*^ cells (Figure S3). Manual annotation of the most down-regulated genes in the *cRFXa*^*PTS–1*^ mutant uncovered a preponderance of genes of putative ciliary function (Figure 3D; File S6). These data indicate that *cRFXa* exerts widespread influence on ciliary gene transcription.

Previous work has shown that animal RFX and FoxJ1 cross-regulate each other’s expression^9,44^. For example, in mouse ependymal cells, RFX3 is required for full *foxJ1* expression^45^, while mouse *foxJ1*^*−/−*^ embryos fail to transcribe *rfx3*^46^. Intriguingly, the most differentially expressed gene in the *S. rosetta cRFXa*^*PTS–1*^ mutant was *foxJ1*, which was 29-fold down-regulated (Figure 3B). This raised the question of whether cRFXa regulates ciliary genes partially through the action of FoxJ1. We found that no single HsaSro conserved ciliary gene was significantly down-regulated in *foxJ1*^*PTS*^ cells (Figure 3B; Files S5, S6). In fact, the only gene significantly differentially expressed in *foxJ1*^*PTS*^ was *trpm3,* which was up-regulated 30-fold in *foxJ1*^*PTS*^ cells (Figure 3B). Together with the observation that ciliogenesis proceeds normally in *foxJ1*^*PTS*^ cells, these data suggest that under standard growth conditions, *foxJ1* is a downstream target of *cRFXa*, but itself has no detectable effect on ciliary gene expression.

Finally, in contrast with the cell cycle regulatory function of RFX in some fungi^24,25^, none of the strongly down-regulated genes in *cRFXa*^*PTS–1*^ mutants had clear connections to cell cycle regulation.

### Predicted RFX binding sites are enriched in promoters of choanoflagellate ciliary genes

**Figure 4.**
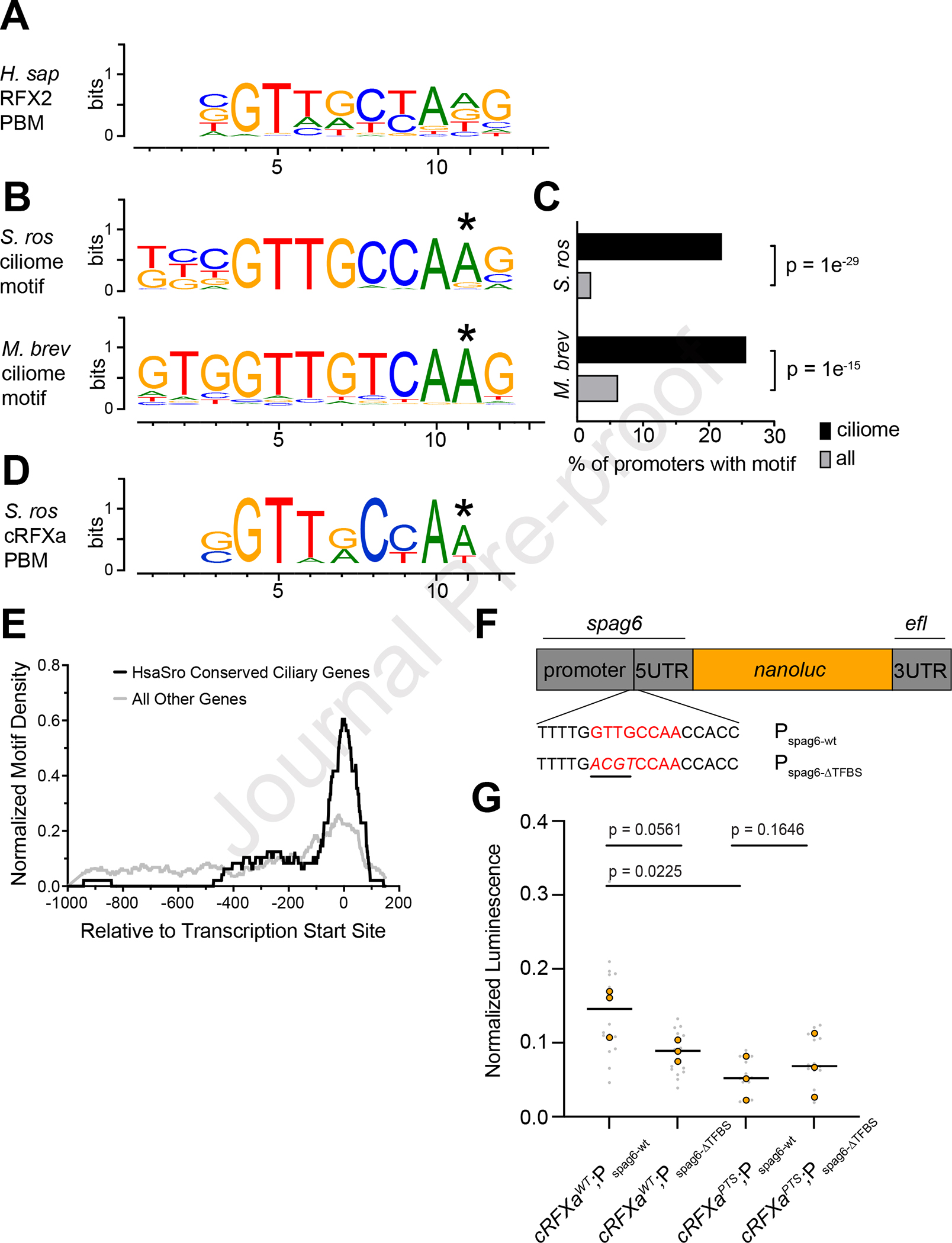
Functional RFX motifs are enriched in choanoflagellate ciliary gene promoters. (A) The *H. sapiens* RFX2 consensus motif as determined by PBM (Cis-BP ID #M02449_2.00)^57^. This motif represents the binding preferences of a single monomer, although RFX binding sites can occur as a tandem inverted repeat of two monomeric sites (the X-box) that bind to an RFX dimer. The DNA binding specificity for RFX TFs is conserved across animal and fungal RFX proteins^10,16,17^. See Figure S4A for an RFX DBD alignment, Figure S4B for the *H. sapiens* RFX2 motif as determined by ChIP-Seq, and Figure S4D for the *M. musculus* FoxJ1 PBM motif. (B) The only enriched sequence motif in the promoters of choanoflagellate ciliary genes matched RFX binding sites from animal studies. Shown are the most enriched HsaSro ciliome promoter motifs for *S. rosetta* and *M. brevicollis*, as determined by the HOMER *de novo* motif finding algorithm. Note the GTTGYCA consensus shared between the two choanoflagellate HsaSro ciliome-enriched motifs and the *H. sapiens* RFX2 motif. This represents the binding specificity of a single RFX DBD. For HOMER, ciliome promoters were defined as 1000 bp upstream and 200 bp downstream of annotated transcription start sites of HsaSro conserved ciliome genes (File S6), although the same RFX motif was recovered using variable definitions of promoter length (Figure S4C). Asterisk indicates a position not shared by animal or fungal RFX motifs. (C) Percentage of HsaSro ciliome promoters with RFX-like motif compared to all mRNA promoters for both *S. rosetta* and *M. brevicollis*. RFX motifs are significantly enriched in HsaSro ciliome promoters compared to all promoters, with enrichment p-values reported by HOMER. (D) The DNA binding specificity of *S. rosetta* cRFXa *in vitro*, as determined by protein binding microarray. The *in vitro* motif was built from the top ten scoring 8-mer hits (E-score range: 0.481–0.486). Asterisk indicates a position not shared by animal RFX motifs. (E) In HsaSro conserved ciliary genes, RFX motifs are preferentially located near transcription start sites. The motif density within promoters is shown for HsaSro conserved ciliome promoters and for all other promoters. The RFX motif identified by HOMER (Figure 4B) in *S. rosetta* was used. Normalized motif density (y-axis) describes the proportion of all motifs that fall into a 100 bp sliding window centered on any given position on the x-axis. The x-axis gives promoter position relative to the predicted transcription start sites of conserved ciliary genes (black line) or all other genes (grey line). See Figure S4E for the same analysis applied to HsaMbrev ciliary promoters using the *M. brevicollis* RFX motif shown in Figure 4B. (F) To functionally test the necessity of predicted an RFX binding site for gene activation, the 5’ UTR and proximal promoter of the *spag6* gene from *S. rosetta* was cloned in front of a *nanoluc* open reading frame which was codon-optimized for *S. rosetta*. A second reporter construct was made in which the predicted RFX binding site was mutated. As an internal normalization step, the plasmid also encodes the *firefly* luciferase under strong expression from the *S. rosetta actin* promoter. (G) Mutation of the predicted RFX binding site in the *spag6* promoter/5’UTR decreased expression of the nanoluc luciferase to an average of 61% of wild-type activity. Three biological replicates were assayed, with 3–6 transfections per construct in each replicate. To normalize for transfection efficiency, the reporter plasmid coded for a second luciferase (*firefly*) under the *actin* promoter. This allowed for the normalization of transfection efficiency by taking the ratio of nanoluc signal to firefly signal. This ratio was then normalized to the expression from the strong *EFL* promoter, included as a positive control in all experiments. Individual values are plotted in gray and averages for each biological replicate plotted in orange. The average across biological replicates is represented by a horizontal bar. P-values are shown for a paired t-test between P_spag6-wt_ and P_spag6-ΔTFBS_ reporters transfected into each genotype, using the mean value for each biological replicate (n = 3), as well as un unpaired t-test for P_spag6-wt_ transfected into either *cRFXa*^*WT*^ or *cRFXa*^*PTS*^ cells, again using the mean value for each biological replicate (n = 3). See also Data S1.

The DNA-contacting residues of RFX DBDs are largely invariant^10,11,47^ (Figure S4A), and the RFX monomeric recognition sequence – GTTRCY – is conserved across fungi and animals^48–51^ (Figure 4A). RFX binding sites often occur as tandem inverted repeats, forming a palindromic sequence referred to as an “X-box”^16,47,49,52^ (GTNRCC N_0–3_ RGYAAC^10^; Figure S4B), which is bound by a dimer of RFX TFs^47,48^. To examine whether RFX might directly regulate ciliary genes in *S. rosetta*, we investigated motif enrichment in the promoters of *S. rosetta* ciliary genes and the DNA binding preferences of cRFXa.

Using the HOMER algorithm^53^, we detected a single motif in *S. rosetta* that was significantly enriched in the promoters of HsaSro conserved ciliome genes (Figure 4B). The motif closely resembles monomeric RFX-bound sequences from humans (Figure 4A) and was detected in 21.9% of promoters from conserved ciliome genes (44 total) as opposed to just 2.0% of all promoters (239 total; Figure 4C). The detected enrichment of the RFX motif in HsaSro conserved ciliome promoters was robust to variable definitions of promoter length (Figure S4C). Out of the 44 HsaSro conserved ciliome genes with RFX motifs, 33 (75%) were significantly down-regulated in *cRFXa*^*PTS*^ cells (File S6). In *M. brevicollis,* the HOMER algorithm also detected an RFX-like motif as the most enriched motif among HsaMbrev conserved ciliome promoters (Figure 4B, C; File S6). In contrast, analysis of conserved ciliome promoters in *Spizellomyces punctatus,* a ciliated chytrid fungus that expresses RFX^54^, did not identify any significantly enriched motifs, RFX or otherwise.

Because the predicted choanoflagellate ciliome motifs matched functionally validated RFX motifs from animals and fungi, we sought to investigate whether cRFXa shares this binding preference. To this end, we used an *in vitro* protein-binding microarray (PBM)^55–57^ in which full-length cRFXa from *S. rosetta* was screened against multiple panels of short DNA oligonucleotides. The consensus motif recovered (Figure 4D) showed clear similarity to both the enriched choanoflagellate ciliome motifs and the binding sites of animal RFX monomers, including those derived from PBM approaches^48,49,57^ (Figure 4A). No similarity to animal FoxJ1 PBM motifs was detected (Figure S4D).

In animals, RFX binding motifs are enriched near transcription start sites^10,58^. We found the same to be true in choanoflagellates, with 60.4% of RFX-like motifs located within 50 bp of the transcription start sites (TSS) of HsaSro conserved ciliary genes (Figure 4E; Figure S4E; File S7). Because we do not know whether choanoflagellates engage in distal regulation of gene transcription, we do not know whether RFX binding motifs detected further from the TSS may still be functional. Interestingly, the *foxJ1* promoter proximal region does not have an RFX binding site meeting our strict criteria, but does have a closely matched sequence (GTTGCGA, compared to the RFX GTTGCCA consensus) 701 base pairs upstream of its transcription start site.

If predicted RFX binding sites are essential for activating transcription of RFX-responsive ciliary genes, disruption of a predicted RFX binding site might be expected to reduce gene transcription. To test this, we focused on the *S. rosetta spag6* ciliary gene, which shows reduced expression in *cRFXa*^*PTS–1*^cells (log_2_FC = −1.50) and has a predicted RFX binding sequence (GTTGCCAA) in its promoter (Figure 4F). We built two reporter constructs: one with *nanoluc* luciferase fused downstream of the wild-type *spag6* promoter (P_spag6-wt_) and a second construct with key nucleotides in the RFX-binding motif mutated from GTTG to ACTG (P_spag6-ΔTFBS_). These constructs were transfected into wild-type and *cRFXa*^*PTS–1*^ cells. Compared to *cRFXa*^*WT*^ cells transfected with the P_spag6-wt_ reporter, *cRFXa*^*PTS–1*^ cells transfected with the P_spag6-wt_ reporter showed reduced *nanoluc* activity (36%; Figure 4G), further implicating cRFXa in the regulation of *spag6*. Furthermore, *cRFXa*^WT^ cells transfected with the P_spag6-ΔTFBS_ reporter showed reduced *nanoluc* activity compared to *cRFXa*^*WT*^ cells transfected with the P_spag6-wt_ reporter (61%; Figure 4G). These results are consistent with the RFX consensus motif being required to mediate full transcription of *spag6*, which can be affected by either mutating the RFX motif or mutating the *cRFXa* gene (Figure 4G).

### The pre-animal ancestry of the RFX ciliogenesis regulatory module

It has previously been unclear whether RFX or FoxJ1 transcription factors regulate ciliogenesis in any non-animal^6,10,11^. One prior study looked for X-box sequences in the promoters of 12 ciliary genes in *M. brevicollis* and suggested that RFX gained control of ciliary genes in animals only after their divergence from choanoflagellates^10^, a conclusion we here revisit in light of increased genomic data and the establishment of transgenics in *S. rosetta*^28,36,59,60^.

We have uncovered four lines of evidence indicating that cRFXa regulates ciliogenesis in *S. rosetta*: (1) targeted disruption of *cRFXa* results in aberrant ciliogenesis; (2) *cRFXa* mutants show significant down-regulation of 93 ciliary genes that are conserved between *S. rosetta* and humans; (3) an unbiased *in silico* approach identified an RFX motif enriched in ciliary gene promoters; (4) an RFX motif is necessary for wild-type levels of gene expression from a ciliary gene promoter.

Disruption of *cRFXa* also results in delayed cell proliferation, which is interesting because RFX homologs regulate the cell cycle in fungi^24,27^. While we did not observe known cell cycle regulators among the most differentially expressed genes in the *cRFXa*^*PTS–1*^ mutant strain, these experiments were not done in synchronized cells, which would allow more sensitive detection of differences in oscillatory gene expression. The defect in cell proliferation may also be due to the ciliogenesis defect, as ciliary function is essential for bacterial prey capture in *S. rosetta*^61^. A defect in prey capture can be seen in our ciliogenesis assay, in which bacteria do not accumulate on the collar until the cilium is fully grown and begins to beat (e.g., time stamp 21:00 in Video S1 and time stamp 47:00 in Video S2 for wild-type cells). In *cRFXa*^*PTS*^ cells that do not assemble cilia in the ciliogenesis assay, bacteria never accumulate on the collar (Videos S3, S4). Therefore, post-mitotic *cRFXa*^*PTS*^ mutant cells may experience nutrient limitation as a secondary consequence of aberrant and delayed ciliogenesis.

Intriguingly, *cRFXa*^*PTS–1*^ cells have steady state ciliary lengths comparable to that of *cRFXa*^*WT*^ cells. This fact, combined with the down-regulation but not total loss of ciliary gene expression (Figure 3B), suggests the presence of other transcriptional regulators of ciliogenesis. These are likely to be factors other than *cRFXb* and *cRFXc*, which were not appreciably transcribed in either *cRFXa*^*WT*^ or *cRFXa*^*PTS–1*^ slow swimmer cells (Figure 1C; File S5).

The comparable roles of *S. rosetta* cRFXa and animal RFX1/2/3 paralogs in regulating ciliogenesis^7,8,19,21^, coupled with the predicted orthology between these two gene sub-families (Figure 1D; also see Chu et al.^11^), suggests that the last common ancestor of animals and choanoflagellates expressed an RFX transcription factor that regulated ciliogenesis. Might the RFX regulatory module be more ancient than the choanoflagellate-animal clade (Choanozoa)? Functional data on ciliated opisthokonts outside the Choanozoa are missing, but our bioinformatic analysis of ciliome promoters in the chytrid *S. punctatus* did not suggest RFX involvement. RFX may have been co-opted to regulate ciliary genes in the Choanozoan stem lineage, perhaps potentiated by RFX family expansion. Alternatively, RFX might have regulated ciliogenesis in stem opisthokonts, but was then recruited for other functions in fungi, including in chytrids. In either scenario, the divergence of RFX functions between choanozoans and fungi required many changes in the *cis*-regulatory sequences of ciliary genes.

### The RFX ciliogenesis regulatory module in the evolution of animal development

One question raised by this work is how the RFX-ciliogenesis regulatory module, likely already present in the protozoan progenitors of choanoflagellates and animals, was integrated into animal developmental programs. Was RFX activity sufficient for specifying ciliated cells, or did it require accessory regulators? If the founders of the modern-day *cRFXa/RFX1/2/3* sub-family had non-ciliogenesis roles, how was pleiotropy resolved when utilizing this network in novel cell type contexts? Finally, the function of FoxJ1 appears to differ in animals and *S. rosetta*. In animals, FoxJ1 regulates many ciliogenesis genes^9^ and shows cross-regulation with RFX. The cross-regulation of these families is also seen in *S. rosetta*, as *foxJ1* is one of the most down-regulated genes upon *cRFXa* disruption. However, disruption of *foxJ1* in *S. rosetta* had no detectable effect on ciliogenesis efficiency and negligible impact on the expression of HsaSro conserved ciliary genes in the slow swimmer cell type. This raises the question of whether FoxJ1 was a sub-module of RFX ancestrally and was later “promoted” to a higher level of the gene regulatory hierarchy or whether the role of FoxJ1 in *S. rosetta* reflects a diminished role from that of its ancestral counterpart.

Finally, our data may add something useful to a growing discussion on the origins of animal cell types. Proposed modes and drivers of cell type evolution include division of labor^62,63^, integration of life cycles^64,65^, stress responses^66^, and gene or genome duplication^67,68^. A common theme in many of these models is the re-purposing of ancestral regulatory connections in novel cell types, in which a single transcription factor can coordinate the activity of a suite of genes sharing complementary functions. The work reported here provides a concrete example of a pre-animal regulatory module, the regulation of which evolved alongside animal development to help differentiate ciliated from non-ciliated cells.

## STAR Methods

### RESOURCE AVAILABILITY

#### Lead contact

Further information and requests for resources and reagents should be directed to and will be fulfilled by the main contact, Nicole King (nking@berkeley.edu)

#### Materials availability

Plasmids generated in this study have been deposited to Addgene (#196406, #196407, #196408).

Choanoflagellate cell lines used in this study are available from the American Type Culture Collection (PRA-390 for wild-type *Salpingoeca rosetta*) or available upon request for mutant cell lines.

#### Data and code availability

RNA sequencing data generated in this study have been deposited to the NCBI Short Read Archive (Project PRJNA91984).

This paper does not report original code. For the use of existing bioinformatic packages, the Method Details specify the options used and the Key Resources Table lists software version numbers.

Any additional information required to reanalyze the data reported in this paper is available from the lead contact upon request.

### EXPERIMENTAL MODEL AND STUDY PARTICIPANT DETAILS

#### Choanoflagellate culture

All experiments used *Salpingoeca rosetta* co-cultured with a single prey bacterial species, *Echinicola pacifica* (ATCC PRA-390, strain designation: SrEpac). Cells were grown in artificial known sea water (AKSW) supplemented with 4% cereal grass media (CGM3) and 4% sea water complete^59^. Cells were grown at 22°C and 60% humidity. For consistency, experiments were done with cells in the mid-log phase of growth, which in this media formulation occurs between 5 × 10^5^ and 3 × 10^6^ cells/ml.

Mutant strains generated by CRISPR/Cas9 genome editing were maintained under the same conditions as wild-type SrEpac, and liquid nitrogen stocks of all generated strains were created. The following mutant lines were generated (see File S4 for editing information and Fig S2A for genotyping traces):

cRFXa^PTS–1^

cRFXa^PTS–2^

cRFXa^REV^

cRFXb^PTS^

cRFXc^PTS^

foxJ1^PTS^

cRFXa^PTS–1^;foxJ1^PTS^

Since the *cRFXb*^*PTS*^, *cRFXc*^*PTS*^, and *foxJ1*^*PTS*^ mutants were generated using a co-editing strategy that confers cycloheximide resistance, the reference wild-type strain for these was SrEpac bearing the P56Q mutation in *rpl36a*^36^.

### METHOD DETAILS

#### BLAST searches for RFX and FoxJ1 genes

To determine the presence of RFX genes throughout eukaryotic diversity, we used a variety of functionally validated RFX DBDs as BLAST queries, searching against the EukProt database, which includes 993 species^28^. First, to define the broad phylogenetic distribution of RFX genes, we queried the DBDs of *Xenopus laevis* RFX2 and *Saccharomyces cerevisiae* RFX1 against the EukProt Comparative Set of 196 species, chosen for taxonomic diversity and genome/transcriptome completeness. EukProt implements the BLASTP 2.13.0 algorithm. We defined bone fide RFX hits as those with at least 75% query coverage and at least 30% sequence identity (see File S1 for DBD probe sequences and EukProt BLAST results).

To develop a comprehensive set of amorphean RFX hits, we used six RFX DBD sequences (*X. laevis* RFX2, *S. cerevisiae* RFX1, *M. musculus* RFX4, *M. musculus* RFX5, *C. elegans* Daf-19, and *S. rosetta* cRFXa) as BLAST probes against a set of 95 amorphean taxa. RFX hits within these taxa were used for the data shown in Fig 1A and to construct the phylogenetic trees in Figure 1D, S1A, S1B, and S1C. All sequences used for phylogenetic tree construction are detailed in File S1. For *S. mediterranea*, which is of interest due to it having demonstrated FoxJ1 function in ciliogenesis^75^, but is not hosted on EukProt, we used the BLASTP server hosted on https://planosphere.stowers.org/, which implements BLASTP 2.3.0.

We used a similar procedure to identify Fox genes, first within the EukProt Comparative Set using the DBDs from *X. laevis* FoxJ1 and *S. mediterranea* FoxJ1 as probes (see File S2 for probe sequences and BLAST results) and a 75% query coverage / 30% query identity threshold criteria. To identify candidate FoxJ1 orthologs for the taxa represented in Figure 1A, reciprocal best BLAST searches were performed, using FoxJ1 DBDs from *M. musculus, X. laevis*, *S. mediterranea*, and *S. rosetta*. For these BLAST searches, we used EukProt for all except two taxa (which are not hosted on EukProt): *S. mediterranea*, hosted at https://planosphere.stowers.org/, and *X. laevis*, for which we used the NCBI BLAST server with the Uniprot reference database. In Figure 1A we report taxa containing reciprocal best BLAST for either *X. laevis* or *S. mediterranea*, which are phylogenetically disparate (within animals) and both have functionally validated FoxJ1 genes with known roles in regulating motile ciliogenesis.

When surveying the distribution of RFX and Fox genes across eukaryotic diversity, our results largely confirmed that RFX genes are widespread among opisthokonts and amoebozoans, while Fox genes are widespread among opisthokonts. However, we did observe rare exceptions to this pattern. Among 539 taxa in EukProt that are not opisthokonts or amoebozoans, three had RFX hits: *Madagascaria erythrocladioides* (a rhodophyte alga), *Gloeochaete wittrockiana* (a glaucophyte alga), and *Siedleckia nematoides* (an alveolate) (File S1). Among 824 non-opisthokonts in EukProt, 14 had Fox hits (File S2). For both the few RFX and Fox hits, the taxa in which they were observed were distributed across eukaryotic diversity. The only obvious pattern was that four out of the eight heterolobosean taxa hosted on EukProt contained Fox hits. Given the rare and dispersed nature of RFX and Fox hits outside of the amoebozoans/opisthokonts and opisthokonts, respectively, we interpret these hits as being more likely due to some combination of horizontal gene transfer, convergent evolution, and possibly sequencing contamination, than due to the presence of RFX or Fox genes in the last common ancestor of eukaryotes.

#### Phylogenetic trees

To build maximum-likelihood trees for RFX family genes, we aligned the protein sequences with MAFFT^76,77^ (v. 7.312) using default options, trimmed with ClipKIT^78^ (v 1.3.0) using the default smart-gap trimming mode, and built trees with IQ-TREE^74^ (v. 2.2.0-beta COVID-edition) using ModelFinder^79^ and 1000 Ultrafast Bootstraps (UF-boot)^80^ or 1000 iterations of SH-aLRT^81^. Trees were visualized with iTOL^82^. To test the robustness of our phylogenetic inferences, alignments were also trimmed with trimAl^83^ (v1.4.rev22) using the gappyout setting and trees were inferred with RAxML^84^ (8.2.11) using the “-f a”, “-m PROTGAMMAAUTO”, and “-N 100” flags to find the best model and perform 100 bootstraps. For IQ-TREE analyses, the best substitution model (as determined by ModelFinder) for the choanoflagellate RFX tree was Q.pfam+F+R5 and for the amorphean RFX tree was Q.pfam+F+R6. For the amorphean RFX tree trimmed with trimAl, the best substitution model was Q.yeast+F+R5.

The protein sequences used for phylogenetic reconstruction are shown in File S1. Note that we do not necessarily use all of the RFX genes within a given taxon, for the purposes of both clarity of presentation and the efficiency of computational bandwidth. This is especially true for vertebrates, with their abundance of RFX duplications within well-established sub-families (e.g. RFX1/2/3 genes), and for some ichthyosporeans (e.g. *C. fragrantissima*), which contain extra RFX genes with long branches that lack consistent placement in phylogenetic re-constructions. These are likely more recent lineage-restricted duplications with extensive divergence.

The only surveyed choanoflagellates without a detectable RFX homolog were uncultured species whose genomes have been sequenced using single-cell technologies^85,86^. These species show relatively lower genome completeness as measured by BUSCO^28,87^. Therefore, the apparent absence of RFX from these species may well be artefactual.

#### RFX DNA-binding domain alignment

For the presentation of RFX DBD alignments in Figure S4A, selected RFX DBD sequences were aligned using MUSCLE^88^ (v. 3.8.425) with a maximum of 8 iterations and all other options as default, implemented in Geneious. However, alignments of full RFX protein sequences were used for the phylogenetic analysis (see previous section on “Phylogenetic Trees” and data in File S1).

#### Choanoflagellate culturing

Unless otherwise specified, all experiments were performed using *Salpingoeca rosetta* co-cultured with a single prey bacterial species: *Echinicola pacifica* (ATCC PRA-390, strain designation: SrEpac). Cells were grown in artificial known sea water (AKSW) supplemented with 4% cereal grass media (CGM3) and 4% sea water complete^59^. Cells are grown at 22°C and 60% humidity. For consistency, experiments were done with cells in the mid-log phase of growth, which in this media formulation occurs between 5 × 10^5^ and 3 × 10^6^ cells/ml.

#### *S. rosetta* cell type RNA sequencing and analysis

Cultures were grown in triplicate for each of four *S. rosetta* cell types. Samples of slow swimmers and rosettes were prepared from cultures of 5% SWC media inoculated with 10^4^ cells/ml of *S. rosetta* feeding on *Echinicola pacifica* bacteria, and rosettes were induced with the addition of outer membrane vesicles (OMVs) from *Algoriphagus machipongonensis*^89^. Both of those cultures were grown for 48 h at 22°C to mid-log phase. Cultures of fast swimmers were inoculated the same as slow swimmers and then grown to starvation for 3 d at 22°C, at which point we transitioned the culture to 30°C for 2.75 h to increase the population of fast swimmers. Thecate cells were prepared by inoculating the HD1 strain of *S. rosetta* – a strain that maintains a higher proportion of thecate cells while also feeding on *E. pacifica* – to 10^4^ cells/ml 10% (v/v) CGM3 and then growing for 48 h at 22°C in square plates.

For each replicate of each cell type, 5 × 10^6^ cells were processed for lysis and RNA extraction. Cells were centrifuged and washed with AKSW. Thecate cells were scraped off the plate first. Cells were resuspended in AKSW, counted, and aliquoted to 10 × 10^6^ per aliquot, then resuspended in 100 μl of lysis buffer^59^: 20 mM Tris-HCl, pH 8.0; 150 mM KCl; 5 mM MgCl_2_; 250 mM sucrose; 1 mM DTT; 10 mM digitonin; 1 mg/mL sodium heparin; 1 mM Pefabloc SC; 100 μg/mL cycloheximide; 0.5 U/μl Turbo DNase; 1 U/μl SUPERaseIN. This was incubated on ice for 10 minutes, passed ten times through a 30G needle and centrifuged at 6,000 × g for 10 minutes at 4°C. The supernatant was collected, brought to 100 μl with RNAse-free water, and RNA was purified using the RNAeasy kit from Qiagen (Cat. No. 74104)., eluting in 30 μl of water.

500 ng were of RNA were used for library prep, first purified with two rounds of polyA mRNA selection with oligo-dT magnetic beads and then converted to sequencing-compatible cDNA using the KAPA mRNA HyperPrep kit (KAPA biosystems, Cat. No. KK8580), using the KAPA single-indexed adapter kit for multiplexing (KAPA biosystems, Cat. No. KK8701). RNA integrity was assessed by Agilent Bioanalyzer 2100 before library prep using an Agilent RNA 6000 Nano Kit (Cat. No. 5067–1511). Sequencing libraries were also confirmed by Bioanalyzer 2100 for the correct size distribution using the Agilent High Sensitivity DNA Kit (Cat. No. 5067–4626). Library concentration was quantified by Qubit and libraries were pooled at equal concentrations before sequencing.

Library sequencing was performed by the QB3-Berkeley Genomics core labs (QB3 Genomics, UC Berkeley, Berkeley, CA, RRID:SCR_022170). Sequencing was performed in one lane on the Illumina HiSeq 4000, collecting between 12.4 million and 61.3 million reads for each sample. Reads were de-multiplexed, checked for quality with fastqc (v 0.11.9), and aligned to predicted transcripts from the *S. rosetta* genome^90^ using Salmon^91^ (v 1.5.2.) and called for differential expression using edgeR^92^, both implemented within the Trinity software package^93^ (v 2.14.0). TPM values for RFX gene expression amongst the different cell stages, as well as differential expression tests comparing slow swimmers with thecate cells, are available in File S3.

#### CRISPR guide RNA and repair template design

Candidate guide RNA sequences were obtained for each gene of interest using the EuPaGDT tool (http://grna.ctegd.uga.edu/) and the *S. rosetta* genome^90^. Guide RNA length was set at 15 and an expanded PAM consensus sequence, HNNRRVGGH, was used. Coding sequences for genes of interest are easily obtained from the Ensembl Protists hosting of the *S. rosetta* genome. Guide RNA candidates were filtered for guides with one on-target hit (including making sure the guides do not span exon-exon boundaries), zero off-target hits (including against the genome of the co-cultured bacterium *E. pacifica*), lowest strength of the predicted secondary structure (assessed using the RNAfold web server: http://rna.tbi.univie.ac.at/cgi-bin/RNAWebSuite/RNAfold.cgi), and annealing near the 5’ end of the targeted gene, particularly before the region encoding the DNA-binding domain. crRNAs with the guide sequence of interest, as well as universal tracrRNAs, were ordered from IDT (Integrated DNA Technologies, Coralville, IA).

Repair templates were designed as single-stranded DNA oligos, in the same sense strand as the guide RNA, with 50 base pairs of genomic sequence on either side of the DSB cut site. Between the homology arms is the TTTATTTAATTAAATAAA insertion cassette. Repair oligos were ordered from IDT as Ultramers.

#### Genome editing

48 h prior to the transfection, *S. rosetta* cells (see File S4 for background genotype of each editing experiment) were inoculated in 120 ml of media at 8,000 cells/ml. This seeding density brings the culture to mid-log phase at the time of transfection. Prior to the day of transfection, dried crRNA and tracrRNA from IDT were each resuspended in duplex buffer (30 mM HEPES-KOH pH 7.5; 100 mM potassium acetate, IDT Cat. No. 11–0103-01) to a concentration of 200 μM. Equal volumes of crRNA and tracrRNA were mixed, incubated for 5 minutes at 95°C in an aluminum heating block, and then cooled to 25°C slowly by removing the heat block from the heating source (with the tube still in it) and cooling to RT. The annealed crRNA/tracrRNA is referred to as the gRNA and can be stored at −20°C for weeks before use. Also prior to the day of transfection, the dried repair oligo was resuspended to 250 μM in 10 mM HEPES-KOH, pH 7.5 and incubated at 55°C for 1 hour, then stored at −20°C.

On the day of transfection, to wash away bacteria from the choanoflagellates, the culture was split into three 50 ml conical tubes and centrifuged for 5 minutes at 2000 × g. The cell pellets were resuspended and combined in 50 ml of AKSW, followed by a 5 min spin at 2200 × g. The cells were washed once more with 50 ml AKSW and spun at 2400 × g. The pellet is resuspended in 100 μl AKSW and diluted 1:100 in AKSW for counting. Cells are diluted to 5 × 10^7^ / mL in AKSW, then 100 μl aliquots (with 5 × 10^6^ cells each) are prepared.

Priming buffer is prepared by diluting 10 μl of 1 mM papain (Sigma-Aldrich Cat. No. P3125–100MG) in 90 μl of dilution buffer (50 mM HEPES-KOH, pH 7.5, 200 mM NaCl, 20% glycerol, 10 mM cysteine, filter-sterilized and stored in aliquots at −80°C). This is then diluted 1:100 in the rest of the priming buffer (40 mM HEPES-KOH, pH 7.5, 34 mM lithium citrate, 50 mM L-cysteine, 15% PEG-8000, filter-sterilized and stored in aliquots at −80°C) for a final concentration of 1 μM papain. The priming buffer can be prepared while washing the cells.

Also while washing the cells, equal volumes of pre-annealed gRNA and *Sp*Cas9 (20 μM, NEB Cat. No. M0646M) are mixed and incubated for 1 h at RT to form the RNP. 4 μl of RNP is used per transfection reaction. The resuspended repair oligo is incubated for 1 hour at 55°C to completely solubilize the material.

Each aliquot of cells is spun at 800 × g for 5 minutes and resuspended in 100 μl priming buffer and incubated for 35 minutes at RT. The priming reaction is quenched by adding 10 μl of 50 mg/ml bovine serum albumin fraction V (Thermo Fisher Scientific Cat. No. BP1600–1000). Cells are spun at 1250 × g for 5 minutes and resuspended in 25 μl Lonza SF buffer (Lonza Cat. No. V4SC-2960) if cycloheximide selection was not used or 200 μl of SF buffer if cycloheximide selection was used.

For each transfection, 16 μl of Lonza SF buffer is mixed with 4 μl of RNP targeting the gene of interest, 2 μl of resuspended repair oligo, and 1 μl of washed and primed cells. If cycloheximide selection is being used, 1 μl of CHX-R RNP is added as well as 0.5 μl of CHX-R repair oligo. These engineer a P56Q mutation in *rpl36a* that confers resistance to cycloheximide^36^. The nucleofection reactions are added to a 96-well nucleofection plate (Lonza Cat. No. V4SC-2960) and pulsed with a CM156 pulse in the Lonza 4D-Nucleofector (Cat. No. AAF-1003B for the core unit and AAF-1003S for the 96-well unit).

After the pulse, 100 μl of ice-cold recovery buffer (10 mM HEPES-KOH, pH 7.5; 0.9 M sorbitol; 8% [wt/vol] PEG 8000) is immediately added to each well of the nucleofection plate and incubated for 5 minutes. Then the entire contents of the well are added to 1 mL of 1.5% SWC + 1.5% CGM3 in AKSW in a 12-well plate and cultured at 22C. After one hour of culture, 10 μl of re-suspended *E. pacifica* bacteria (10 mg/ml in 1 ml AKSW) are added to each culture not undergoing cycloheximide selection, and 50 μl are added for each culture that is undergoing cycloheximide selection.

The following day, 10 μl of 1 ug/ml cycloheximide is added to wells undergoing cycloheximide selection. Selection was done for 4 days.

Clonal dilutions were done 24 hours after transfection for cells not undergoing cycloheximide selection, and 5 days after transfection (with 4 days of selection) for cells undergoing cycloheximide selection. Cells were counted and diluted to 2 cells/ml in 1.5% SWC + 1.5% CGM3 in AKSW. To this was added a 1:1000 dilution of re-suspended *E. pacifica* (10 mg/ml in 1 ml AKSW). 200 μl of diluted culture was added per well for 96-well plates. For each editing experiment, between 5 and 20 96-well plates were prepared.

To genotype, 96-well plates were screened by microscopy and wells containing choanoflagellates were marked. These were re-arrayed into fresh 96-well plates with each well containing a separate clone. To extract genomic DNA, 50 μl of cell culture was mixed with 50 μl of DNAzol direct (Molecular Research Center, Inc [MRC, Inc.], Cincinnati, OH; Cat. No. DN131), incubated at RT for 10 minutes and stored at −20°C. Genotyping PCRs were performed in 96-well plates (Brooks Life Sciences Cat. No. 4ti-0770/c) using Q5 polymerase (NEB Cat. No. M0491L), and 40 cycles of amplification. 5 μl of genomic DNA template were used in a 50 μl PCR reaction. PCR products were purified by magnetic bead clean-up and were analyzed by Sanger sequencing (UC Berkeley DNA Sequencing Facility).

#### Measuring ciliary lengths

To measure cilium length, cells grown to mid-log phase were fixed and stained using 1 part Lugol’s solution (EMD Millipore Cat. No. 1.09261.1000) with 3 parts culture (usually 25 μl and 75 μl). 4 μl were loaded onto a slide, spread by placing a No. 1.5 coverslip on thee sample, and imaged coverslip slide down with a Zeiss Axio Observer.Z1/7 Widefield microscope with a Hamamatsu Orca-Flash 4.0 LT CMOS Digital Camera (Hamamatsu Photonics, Hamamatsu City, Japan) and 40×/NA 1.1 LD C-Apochromatic water immersion objective. Images were acquired with 10 ms exposure and 8.0 V of light intensity, using the PH3 phase contrast ring. Ciliary lengths were traced and measured in Fiji^94^.

#### Genome editing for *cRFXa* revertant

To revert the *cRFXa*^*PTS–1*^ strain to a wild-type amino acid sequence, we transfected Cas9 with guide RNAs that cut on either side of the PTS allele and included a repair template that introduces a GTC > GTG (Valine) synonymous mutation in the wild-type gene sequence, allowing us to distinguish revertants from wild-type cells by genotyping. We first transfected various single and dual gRNA combinations into the *cRFXa*^*PTS–1*^ strain and assessed editing frequency by next-generation amplicon sequencing 24 hours post-transfection. To do this we extracted DNA as in the “Genome Editing” section, PCR amplified around the PTS insertion using primers TGTCATGTTCTTTGCTGGCG and GTCGAAGGCGTTGAAGTTGC, and submitted purified PCR products for Genewiz Amplicon-EZ services (Azenta Life Sciences, Chelmsford, MA). Editing efficiency was very low for all gRNAs tested, with a maximum of 0.04% for the combination listed in File S4. This may be due to using an NGG PAM instead of the stricter HNNRRVGGH PAM^36^, which had no consensus sites near the PTS insertion.

Despite the low efficiency, we reasoned that due to the growth defect of the *cRFXa*^*PTS*^ mutant, a revertant might out-compete non-reverted cells in a mixed population. To test this, we cultured the transfected cultures for 4 weeks, isolated clones, and genotyped the locus. All genotyped clones were had the reverted allele, showing the success of this competition strategy.

#### Ciliogenesis assay

For step-by-step protocol, see protocols.io: dx.doi.org/10.17504/protocols.io.q26g7y9n3gwz/v2

To monitor ciliogenesis, cells were grown to mid-log phase, counted, and 6 × 10^6^ cells were centrifuged in a 15 ml falcon tube for 10 minutes at 2000 × g. The cell pellet was resuspended in 1 ml of 90% AKSW / 10% glycerol, added to a FluoroDish (World Precision Instruments Cat. No. FD35–100) and incubated for 7 minutes at −20°C. This method of ciliary removal was inspired by a ciliary removal protocol from *Chlamydomonas*^37^. For *S. rosetta*, we observed that on average 85% of cells lost their cilium, with a range of 68%–98%. A second FluoroDish was treated with 10 seconds of corona discharge (Electro-Technic Products BD-20AC), then rinsed with 1 ml of 0.1 mg/ml poly-D-Lysine (Millipore Sigma Cat. No. P6407–5MG). The dish was rinsed 3x with water and air dried.

After incubation at −20°C, the cells were transferred to a 1.5 ml Eppendorf tube and spun for 10 minutes at 4200 × g. The cell pellet was resuspended in 25 μl AKSW and transferred to the lysine-coated FluoroDish. A 22 mm circular diameter #1.5 coverslip (Electron Microscopy Sciences Cat No. 72224–01) was gently laid on top. The dish was positioned on the microscope stage and after the cells were brought into focus, the dish was flooded with 1 mL of AKSW to dislodge the coverslip while leaving the cells stuck to the surface. Cells were imaged with a Zeiss Axio Observer.Z1/7 Widefield microscope with a Hamamatsu Orca-Flash 4.0 LT CMOS Digital Camera (Hamamatsu Photonics, Hamamatsu City, Japan) and 100 × NA 1.40 Plan-Apochromatic oil immersion objective (Zeiss) using a differential interference contrast (DIC) filter. Images were acquired at 10 z-slices spanning 10 μm, with one stack acquired every 30 seconds for one hour. We used 12.2 V bulb intensity and a short exposure (5 ms) to best capture the position of the flagellum as it regrew.

Image analysis was done in Fiji, marking the time point at which ciliogenesis was complete. This was defined as the point at which the growing cilium crossed the outer edge of the microvillar collar. In cases where the microvillar collar was significantly shortened by the glycerol treatment, the collar was able to re-lengthen quickly, almost always faster than the pace of ciliary re-generation. The time point at which the cilium crossed the microvillar collar could be assessed by DIC microscopy, while exact ciliary lengths were hard to extrapolate from live cells, due to ciliary motion and the various angles at which cells were oriented relative to the imaging plane. Cells were excluded from analysis for the following reasons: if it was impossible to determine when or whether the cilium crossed the outer edge of the microvillar collar; if the cell still maintained a cilium at time 0 (occasionally a nub of a cilium had already started to regenerate by the time the cells were put on the microscope, so a pre-existing cilium was defined as a cilium greater than 2 μm in length); if the cell divided or fused with a nearby cell during the time-course; if a cell contained multiple cilia (due to fusion or incomplete cytokinesis); if the cell was obviously dead (this could be diagnosed by the cell having irreversibly lost its microvillar collar and not making any attempts to regenerate the cilium or collar).

#### Growth curves

Cells in mid-log phase were diluted to 5,000 / ml and supplemented with 10 μg/ml *E. pacifica* bacteria (diluted 1:1000 from a stock of 10 mg/ml in AKSW). 500 μl of culture was aliquoted into each well of a 24-well plate (Fisher Scientific Cat. No. 09–761-146) and cultured at 22°C. Plates were kept in a Tupperware box with dampened paper towels and the lid loosely affixed to prevent cultures from drying out but to allow gas exchange.

Every 12 hours for 96 hours, 3 wells from each strain were fixed with 10 μl of 16% paraformaldehyde (Fisher Scientific Cat. No. 50–980-487) and stored at 4°C. After all time points were collected, each sample was counted by vortexing the sample at high speed for 10 seconds to fully mix the sample, then aliquoting 10 μl into a counting slide (Logos Biosystems Cat. No. L12001 [disposable] or L12011 [reusable]) and counting using a Luna-FL automated cell counter (Logos Biosystems, Anyang, KOR; Cat. No. L20001).

#### RNA sequencing and differential expression analysis for cRFXa and FoxJ1 mutants

30 ml of cells were grown to mid-log phase. For *cRFXa*^*PTS–1*^, wild-type *S. rosetta* was used as the wild-type comparison strain. For *foxJ1*^*PTS*^, which was isolated using cycloheximide resistance selection and contains the co-edited *rpl36a*^*P56Q*^ allele, the wild-type comparison strain was a clone with only the *rpl36a*^*P56Q*^ mutation^36^. Three biological replicates were prepared, each on a separate day, processing one wild-type and one mutant culture at a time for cell lysis and RNA extraction.

For each replicate of each strain, 5 × 10^6^ cells were processed for lysis and RNA extraction. Cells were centrifuged and washed with AKSW. Cells were resuspended in AKSW, counted, and aliquoted to 10 × 10^6^ per aliquot, then resuspended in 100 μl of lysis buffer. This was incubated on ice for 10 minutes, passed ten times through a 30G needle and centrifuged at 6,000 × g for 10 minutes at 4°C. The supernatant was collected, brought to 100 μl with RNAse-free water, and RNA was purified using the RNAeasy kit from Qiagen, eluting in 30 μl of water (Cat. No. 74104).

Library preparation and sequencing was performed by the QB3-Berkeley Genomics core labs (QB3 Genomics, UC Berkeley, Berkeley, CA, RRID:SCR_022170). 500 ng were of RNA were used for library prep using the KAPA mRNA capture kit (Cat. No. 07962240001) for poly-A selection and the KAPA RNA HyperPrep kit (Cat. No. 08105952001). Truncated universal stub adapters were ligated to cDNA fragments, which were then extended via PCR using unique dual indexing primers into full length Illumina adapters. RNA integrity was assessed by Agilent Bioanalyzer 2100 before library prep using an Agilent RNA 6000 Nano Kit (Cat. No. 5067–1511). Sequencing libraries were also confirmed by Bioanalyzer 2100 for the correct size distribution using the Agilent High Sensitivity DNA Kit (Cat. No. 5067–4626). Library concentration was quantified by qPCR using the KAPA Library Quantification Kit (Cat. No. 079601400001) and libraries were pooled at equal concentrations before sequencing.

Sequencing was performed in one lane of an SP flow cell on the Illumina NovaSeq 6000 with an S4 flowcell, collecting between 45.4 million and 73.3 million 50 bp paired-end reads for each sample. Reads were de-multiplexed using Illumina bcl2fastq2 (v 2.20) and default settings, on a server running CentOS Linux 7. Reads checked for quality with fastqc (v 0.11.9), and aligned to predicted transcripts from the *S. rosetta* genome^90^ using Salmon^91^ (v 1.5.2.) and called for differential expression using edgeR^92^, both implemented within the Trinity software package^93^ (v 2.14.0). Transcripts with an average TPM value less than 1 for both wild-type and mutant cells were excluded from analysis. Further analysis and comparisons were done using Python scripts in Jupyter Notebook with plotting in Prism 9. TPM values for all replicates and differential expression tests are shared in File S5.

#### Conserved ciliome genes

Lists of evolutionarily conserved ciliary genes have been assembled by comparing datasets across eukaryotic diversity using approaches such as comparative genomics and mass spectrometry. Previous compilations of ciliary genes have been published as the Ciliary proteome database^95^, Cildb^96^ and SYSCILIA^97^.

Building on these databases, we curated our own set of human ciliary genes, focusing on components with a described functional role in ciliogenesis (File S6). Our list contained 269 genes. We identified likely orthologs of these genes in *S. rosetta, M. brevicollis,* or *S. punctatus* using the criteria of reciprocal best BLAST hits or a BLAST e-value < 1e^−20^. Finally, we removed duplicate hits to finalize a list of conserved ciliary genes, which was used for downstream analysis of RNA sequencing data and promoter motif content. 201 human ciliary genes were conserved in *S. rosetta*, 176 in *M. brevicollis*, and 182 in *S. punctatus*.

#### Protein binding microarray

RNA was prepared from wild-type *S. rosetta* cells grown to mid-log phase using the methods for lysis and RNA extraction described previously (see: *S. rosetta* cell type RNA sequencing and analysis). cDNA was prepared form this RNA using the SuperScript IV reverse transcriptase kit (Thermo Fisher Scientific, Cat. No. 18091050), with 150 ng of RNA input and dT(20) primers. The cRFXa CDS was amplified from cDNA using primers ATGTCACAGCAACAGGGGGT and CACGTCCGGTGGCCG using Q5 DNA polymerase (NEB Cat. No. M0491L), with 2 μl of cDNA template in a 50 μl PCR reaction and 35x cycles. The PCR product was gel purified (Qiagen, Venlo, NLD, Cat. No. 28706) and cloned into TOPO pCR2.1 (Thermo Fisher Scientific Cat. No. K450001) after A-tailing with Taq polymerase (NEB Cat. No. M0273S) for 15 minutes at 72°C. The TOPO reaction was transformed into TOPO OneShot cells, cultured over-night, mini-prepped (Qiagen, Cat. No. 27106) and confirmed for correct insertion with Sanger sequencing (UC Berkeley DNA Sequencing Facility) using M13R primer.

The cRFXa CDS was amplified from the TOPO vector using primers TGCAGAGCTCAGGCGCGCCATGTCACAGCAACAGGGGGT and GCCGGATCCTCACCTGCAGGTCACGTCCGGTGGCCG using Q5 DNA polymerase in a 50 μl PCR reaction. The primers contain homology arms for Gibson assembly into the pTH6838 vector, which was linearized with restriction enzyme XhoI (NEB Cat. No. R016S). The pTH6838 vector is a T7-driven expression vector with a N-terminal GST tag. The amplified CDS and XhoI-digested vector were gel purified. Gibson assemblies were performed using the NEB HiFi Assembly Kit (New England Biolabs, Cat. No. E2621L) with 100 ng of insert and a 2:1 molar ratio of insert:vector. The Gibson reaction was transformed into chemically competent XL10 Gold *E. coli* (Agilent, Santa Clara, CA, Cat. No. 200315), cultured over-night, mini-prepped and confirmed for correct insertion with Sanger sequencing.

The TF samples were expressed by using a PURExpress In Vitro Protein Synthesis Kit (New England BioLabs) and analyzed in duplicate on two different PBM arrays (HK and ME) with differing probe sequences. PBM laboratory methods including data analysis followed the procedure described previously^55,56^. PBM data were generated with motifs derived using Top10AlignZ^57^.

#### Promoter transcription factor motif analysis

From the conserved ciliary genes in *S. rosetta*, *M. brevicollis,* or *S. punctatus* (File S6), we extracted the promoter regions, defined as 1000 base pairs upstream and 200 base pairs downstream of annotated transcription start sites, although other promoter definitions were tested to ascertain the robustness of the results (Figure S4C). Using the ciliary promoters and a background set of all promoters (−1000 to 200 bp from all protein-coding genes), we looked for ciliome-enriched motifs using HOMER^53^, specifically the findMotifs.pl script with default options. To create a list of motif instances from a HOMER-identified motif, we also called findMotifs.pl with the -find option.

For *S. rosetta*, we used gene models from assembly Proterospongia_sp_ATCC50818, hosted on Ensembl Protist. For *M. brevicollis*, we used gene models from assembly GCA_000002865.1, hosted on Ensembl Protist. For *S. punctatus*, we used gene models from assembly DAOM BR117, hosted on Ensembl Fungi.

#### Luciferase Reporter Assays

To compare luciferase activity between promoters, we built plasmids expressing both nanoluc and firefly luciferases codon-optimized for *S. rosetta*. This allows one promoter to be variable between plasmids while keeping the other promoter constant as a control for efficiency of transfection and plasmid retention. A codon-optimized *nanoluc* was previously published^59^; therefore we ordered a codon-optimized *firefly* as a gBlock (Integrated DNA Technologies) and ligated this in between 5’ and 3’ regulatory regions of *S. rosetta actin* (XM_004993513.1) in the NK587 backbone (Addgene), creating a new plasmid called NK621 (Addgene).

To construct the dual-luciferase plasmid, a fragment containing the *S. rosetta efl* (XM_004996684.1) 5’ and 3’ regulatory regions flanking the *nanoluc* ORF was digested from plasmid NK606 (Addgene) using MfeI-HF (Cat. No. R3589S) and KpnI-HF (Cat. No. R3142S) restriction enzymes from New England Biolabs. NK809, containing the *S. rosetta actin* (XM_004993513.1) 5’ and 3’ regulatory regions flanking the *firefly* ORF, was linearized using KpnI-HF (Cat. No. R3142S), EcoRI-HF (Cat. No. R3101S), and CIP (M0290S) from New England Biolabs. The fragments were purified on a 1% agarose gel and extracted with QIAquick Gel Extraction Kit (Qiagen Cat. No. 28706). The purified fragments were ligated using the Roche Rapid DNA Ligation Kit (Roche Diagnostics Cat. No. 11635379001) using 90 ng of total DNA and 5:1 ratio of insert:vector, then transformed into chemically competent XL10 Gold *E. coli* (Agilent, Santa Clara, CA, Cat. No. 200315), cultured over-night, mini-prepped and confirmed for correct assembly with Sanger sequencing. The resulting plasmid is identified as NK809 (Addgene #196406).

To test different promoters with this reporter plasmid, the 5’ *efl* region next to *nanoluc* was replaced with a 5’UTR/promoter of interest. From *S. rosetta* genomic DNA, the 5’ upstream region of the *spag6* gene (XM_004991453.1) including the 133 bp annotated 5’ UTR plus an additional 852 bp upstream of that were amplified using forward primer CTCACTCATTCTCTGCTGC and reverse primer CTTGTCTGTTTCGTGTGTGTG using Q5 DNA polymerase (NEB Cat. No. M0491L) in a 50 μl PCR reaction with 35x cycles. This was gel purified (Qiagen Cat. No. 28706) and cloned into TOPO pCR2.1 (Thermo Fisher Scientific Cat. No. K450001) after A-tailing with Taq polymerase (NEB Cat. No. M0273S) for 15 minutes at 72°C. The TOPO reaction was transformed into TOPO OneShot cells, cultured over-night, mini-prepped (Qiagen, Cat. No. 27106) and confirmed for correct insertion with Sanger sequencing (UC Berkeley DNA Sequencing Facility) using M13R primer. The NK809 backbone was amplified to include everything except for the *pEFL* sequence using primers TGCAAATTGTACAGAAGTCACTGT and ATGTCTGTCTTCACCCTCG using Q5 DNA polymerase. A minimal *spag6* promoter containing the 133 bp 5’ UTR and 138 bp of additional 5’ sequence was amplified to include homology arms for pMC001 without *pEFL* using primers ACTTCTGTACAATTTGCAAGACAACGCGCTGAAGAAGA and GAGGGTGAAGACAGACATCTTGTCTGTTTCGTGTGTGTGT. These two PCR products were ligated in a Gibson assembly using the NEB HiFi Assembly Kit (New England Biolabs, Cat. No. E2621L) with 100 ng of insert and a 2:1 molar ratio of insert:vector. The Gibson reaction was transformed into chemically competent XL10 Gold *E. coli* (Agilent, Santa Clara, CA, Cat. No. 200315), cultured over-night, mini-prepped and confirmed for correct insertion with Sanger sequencing. The resulting plasmid is called NK810 (Addgene #196407).

To mutate the RFX binding site in the *spag6* regulatory region, from GTTGCCAA to ACGTCCAA, the SPAG6 plasmid was amplified using primers TGTTGGCGTTGGCGGTGGTTGGACGTCAAAACAACGAAAATTACCCCAAATC and GATTTGGGGTAATTTTCGTTGTTTTGACGTCCAACCACCGCCAACGCCAACA, then assembled using the Agilent QuikChange Lightning Side-Directed Mutagenesis Kit (Cat. No. 210518), using DpnI to degrade the methylated (and non-mutated) template backbone. The reaction was transformed into chemically competent XL10 Gold *E. coli* (Agilent, Santa Clara, CA, Cat. No. 200315), cultured over-night, mini-prepped and confirmed for correct insertion with Sanger sequencing. The resulting plasmid is called NK811 (Addgene #196408).

To prepare for plasmid transfection into *S. rosetta*, the NK809, NK810, and NK811 plasmids were transformed into dam-/dcm- *E. coli* (New England Biolabs Cat. No. C2925H), then sent for large-scale preps and concentration to a value of 10 μg/μl in 10 mM Tris pH 8.5 using the Genewiz service (Azenta Life Sciences, Chelmsford, MA).

The plasmids were transfected into *S. rosetta* using the following protocol, which is similar to the genome editing protocol with some important differences. 48 h prior to the transfection, *S. rosetta* cells were inoculated in 120 ml of media at 8,000 cells/mL. This seeding density brings the culture to mid-log phase at the time of transfection.

On the day of transfection, to wash away bacteria from the choanoflagellates, the culture was split into three 50 ml conical tubes and centrifuged for 5 minutes at 2000 × g. The cell pellets were resuspended and combined in 50 ml of AKSW, followed by a 5 min spin at 2200 × g. The cells were washed once more with 50 ml AKSW and spun at 2400 × g. The pellet is resuspended in 100 μl AKSW and diluted 1:100 in AKSW for counting. Cells are diluted to 5 × 10^7^ / mL in AKSW, then 100 μl aliquots (with 5 × 10^6^ cells each) are prepared.

Priming buffer is prepared by diluting 10 μl of 1 mM papain (Sigma-Aldrich Cat. No. P3125–100MG) in 90 μl of dilution buffer (50 mM HEPES-KOH, pH 7.5, 200 mM NaCl, 20% glycerol, 10 mM cysteine, filter-sterilized and stored in aliquots at −80°C). This is then diluted 1:67 in the rest of the priming buffer (40 mM HEPES-KOH, pH 7.5, 34 mM lithium citrate, 50 mM L-cysteine, 15% PEG-8000, filter-sterilized and stored in aliquots at −80°C) for a final concentration of 1.5 μM papain (compared to 1 μM papain for genome editing). The priming buffer can be prepared while washing the cells.

Each aliquot of cells is spun at 800 × g for 5 minutes and resuspended in 100 μl priming buffer and incubated for 35 minutes at RT. The priming reaction is quenched by adding 1 μl of 50 mg/ml bovine serum albumin fraction V (Thermo Fisher Scientific Cat. No. BP1600–1000). Cells are spun at 1250 × g for 5 minutes and resuspended in 25 μl Lonza SF buffer (Lonza Cat. No. V4SC-2960).

For each transfection, 16 μl of Lonza SF buffer is mixed 1 μl of 10 μg/μl plasmid, 1 μl of 10 mM Tris pH 8.5, 2 μl of re-suspended *S. rosetta* cells, and 4 μl of the plasmid nucleofection master mix (10 μg/μl pUC19 plasmid DNA, 62.5 mM ATP-NaOH pH 7.5, 25 mg/ml heparin).

The nucleofection reactions are added to a 96-well nucleofection plate (Lonza Cat. No. V4SC-2960) and pulsed with a CM156 pulse in the Lonza 4D-Nucleofector (Cat. No. AAF-1003B for the core unit and AAF-1003S for the 96-well unit).

After the pulse, 100 μl of ice-cold recovery buffer (10 mM HEPES-KOH, pH 7.5; 0.9 M sorbitol; 8% [wt/vol] PEG 8000) is immediately added to each well of the nucleofection plate and incubated for 5 minutes. Then the entire contents of the well are added to 1 mL of 1.5% SWC + 1.5% CGM3 in AKSW in a 12-well plate and cultured at 22C. After one hour of culture, 10 μl of re-suspended *E. pacifica* bacteria (10 mg/ml in 1 ml AKSW).

24 hours after transfection, cells were prepared for the reporter assay. For each sample, the 1 ml culture was centrifuged at 4200 × g for 15 mins at 4C. The cell pellet was resuspended in 50 μl of lysis buffer [50 mM HEPES, pH 7.6, 100 mM NaCl, 1% (v/v) Triton X-100, 2 mM Pefabloc, 1 Roche EDTA free complete mini/5 mL, 1 mM EDTA, 10% (v/v) glycerol, 2 mM DTT], transferred to a white flat-bottom 96-well plate (Greiner Bio-One Cat. No. 655083) and incubated at RT for 10 mins. The lysates were analyzed for luciferase activity using the Nano-Glo Dual-Luciferase Reporter Assay System (Promega Cat. No. N1610). Luminescence was read on the SpectraMax i3x plate reader (Molecular Devices), using photon counting with 1 second of integration.

### QUANTIFICATION AND STATISTICAL ANALYSIS

Information about the quantification and statistical details of experiments can be found in the corresponding figure legends. Statistical tests and graphs were produced using Prism 9.0.0.

## Supplementary Material

Data S1 Data S1. Supporting information for phylogenetics, gene editing, RNA sequencing, conserved ciliary genes, and RFX motifs. Related to Figures 1–4.
File S1 contains the query sequences used to identify RFX family members by BLAST (Tab 1), as well as detailed BLAST results (Tabs 5–15). It also includes the sequences used for both choanoflagellate and opisthokont/amoebozoan RFX phylogenetic tree construction (Tabs 3–4), as well as a summary of which choanoflagellate species contain which RFX sub-families (Tab 2).
File S2 contains the query sequences used to identify Fox family members and candidate FoxJ1 orthologs by BLAST (Tab 1), as well as detailed BLAST results (Tabs 2–7). It also includes a summary of reciprocal best BLAST analysis for all taxa shown in Figure 1A (Tab 8)
File S3 shows TPM values for *cRFXa*, *cRFXb*, *cRFXc*, and *foxJ1* expression in each of three biological replicates across slow swimmer, fast swimmer, rosette, and thecate life history stages (Columns C-N). It also shows the results of edgeR differential expression tests comparing slow swimmer to thecate expression for each gene (Columns O-Q).
File S4 contains detailed information on all CRISPR gene editing experiments, including gRNA sequences (Column C), homology-directed repair template sequences (Column D), and the number of clones genotyped (Column H).
File S5 shows edgeR differential expression results for both *cRFXa*^*PTS–1*^ (Tab 1) and *foxJ1*^*PTS*^ (Tab 3) RNA expression compared to wild-type controls. Only included are transcripts that showed an average TPM > 1 in at least one genotype. In addition to differential expression results, genes are annotated with their predicted InterProScan^98^ domains (Column E), the closest human BLAST hit (including name and BLAST result statistics; Columns F-K), and whether the protein product of the transcript was detected by mass spectrometry in *S. rosetta* cilia (Columns L-M). Separate tabs show raw TPM values across all biological replicates (Tab 2 for *cRFXa*, Tab 4 for *foxJ1)*.
File S6 shows the BLAST results of a core set of human ciliary genes (Tabs 1–2, Columns A-F) queried against *S. rosetta* (Tab 1, Columns G-M), *M. brevicollis* (Tab 1, Columns N-T), and *S. punctatus* (Tab 2, Columns G-M), including whether the listed BLAST hit represents a reciprocal best hit. For the HsaSro conserved ciliary set, Tab 3 summarizes both *cRFXa* and *foxJ1* differential expression data, and indicates the presence or absence of a high-confidence RFX motif in the associated promoter region (Col N). Finally, for all genes strongly down-regulated (log_2_FC < −2) in the *cRFXa*^*PTS–1*^ strain, this file shows the InterProScan annotations (Tab 4, Col E), best human BLAST hit (Tab 4, Col F-G), and a manual categorical annotation (e.g. cilium, ion channel, unknown; Tab 4, Col L).
File S7 shows all high-scoring matches to ciliome-enriched RFX motifs identified from *S. rosetta* (Tab 1) and *M. brevicollis* (Tab 2), across all genome-wide annotated promoters, using the HOMER findMotifs.pl script. A column also shows whether the transcript associated with each motif is part of the conserved ciliome with *H. sapiens* (Column H).

Supplementary Figures

Video S1 Video S1. Ciliogenesis of *cRFXa*^*WT*^, example 1. Related to Figure 2E, G, H.
A time-lapse video of a *cRFXa*^*WT*^ cell for 60 minutes after losing its cilium (frames every 30 seconds). The regeneration process can be seen as a new cilium extends from the apical end of the cell. Scale bar = 5 μm.

Video S2 Video S2. Ciliogenesis of *cRFXa*^*WT*^, example 2. Related to Figure 2E, G, H.
A time-lapse video of a *cRFXa*^*WT*^ cell for 60 minutes after losing its cilium (frames every 30 seconds). The regeneration process can be seen as a new cilium extends from the apical end of the cell. Scale bar = 5 μm.

Video S3 Video S3. Ciliogenesis of *cRFXa*^*PTS–1*^, example 1. Related to Figure 2F, G, H.
A time-lapse video of a *cRFXa*^*PTS–1*^ cell for 60 minutes after losing its cilium (frames every 30 seconds). The regeneration process can be seen as a new cilium extends from the apical end of the cell. Scale bar = 5 μm.

Video S4 Video S4. Ciliogenesis of *cRFXa*^*PTS–1*^, example 2. Related to Figure 2F, G, H.
A time-lapse video of a *cRFXa*^*PTS–1*^ cell for 60 minutes after losing its cilium (frames every 30 seconds). The regeneration process can be seen as a new cilium extends from the apical end of the cell. Scale bar = 5 μm.

## Figures and Tables

**KEY RESOURCES TABLE:** 

REAGENT or RESOURCE	SOURCE	IDENTIFIER
Bacterial and Virus Strains		
*E. coli* XL10 Gold competent cells	Agilent Technologies	Cat #200315
*E. coli* dam-/dcm- competent cells	New England Biolabs	Cat #C2925H
Chemicals, Peptides, and Recombinant Proteins		
Engen Cas9 NLS *S. pyogenes*	New England Biolabs	Cat #M0646M
Duplex Buffer	Integrated DNA Technologies	Cat #11–0103-01
Cycloheximide	Sigma Aldrich	Cat #C7698–5G
Lugol’s solution	EMD Millipore	Cat #1.09261.1000
Papain	Sigma Aldrich	Cat #P3125–100MG
DNAzol direct	Molecular Research Center	Cat #DN131
16% paraformaldehyde	Fisher Scientific	Cat #50–980-487
poly-D-lysine	Millipore Sigma	Cat #P6407–5MG
Critical Commercial Assays		
Nano-Glo Dual-Luciferase Reporter Assay	Promega	Cat #N1610
KAPA mRNA HyperPrep Kit	Kapa Biosystems	Cat #KK8580
KAPA mRNA Capture Kit	Kapa Biosystems	Cat #07962240001
HiFi Assembly Kit	New England Biolabs	Cat #E2621L
QuikChange Lightning Side-Directed Mutagenesis Kit	Agilent Technologies	Cat #210518
Rapid DNA Ligation Kit	Roche Diagnostics	Cat #11635379001
Deposited Data		
RNA sequencing data	This paper	NCBI Short Read Archive Project #PRJNA91984
Experimental Models: Organisms/Strains		
*Salpingoeca rosetta* choanoflagellate co-cultured with *Echinicola pacifica* (SrEpac)	American Type Culture Collection	Cat #PRA-390
Genome-edited SrEpac: *cRFXa*^*PTS-1*^*, cRFXa*^*PTS-2*^*, cRFXa*^*REV*^*, cRFXb*^*PTS*^*, cRFXc*^*PTS*^*, foxJ1*^*PTS*^*, cRFXa*^*PTS-1*^*;foxJ1*^*PTS*^	This paper	
Oligonucleotides		
Guide RNAs and oligonucleotide repair templates	This paper, synthesized by Integrated DNA Technologies	Data S1
Amplify cRFXa CDS from cDNA: ATGTCACAGCAACAGGGGGT (Forward) and CACGTCCGGTGGCCG (Reverse	This paper, synthesized by Integrated DNA Technologies	File S4
Amplify cRFXa CDS from TOPO vector for Gibson assembly cloning into PBM expression vector: TGCAGAGCTCAGGCGCGCCATGTCACAGCAACAGGGGGT (Forward) and GCCGGATCCTCACCTGCAGGTCACGTCCGGTGGCCG (Reverse)	This paper, synthesized by Integrated DNA Technologies	N/A
Amplify *spag6* promoter region from genomic DNA: ACTCACTCATTCTCTGCTGC (Forward) and CTTGTCTGTTTCGTGTGTGTG (Reverse)	This paper, synthesized by Integrated DNA Technologies	N/A
Amplify NK809 backbone without pEFL: TGCAAATTGTACAGAAGTCACTGT (Forward) and ATGTCTGTCTTCACCCTCG (Reverse)	This paper, synthesized by Integrated DNA Technologies	N/A
Gibson assembly of *spag6* promoter into NK809 without pEFL: ACTTCTGTACAATTTGCAAGACAACGCGCTGAAGAAGA (Forward) and GAGGGTGAAGACAGACATCTTGTCTGTTTCGTGTGTGTGT (Reverse)	This paper, synthesized by Integrated DNA Technologies	N/A
Site-directed mutagenesis of *spag6* promoter in NK810: TGTTGGCGTTGGCGGTGGTTGGACGTCAAAACAACGAAAATTACCCCAAATC (Forward) and GATTTGGGGTAATTTTCGTTGTTTTGACGTCCAACCACCGCCAACGCCAACA (Reverse)	This paper, synthesized by Integrated DNA Technologies	N/A
tracrRNA	Integrated DNA Technologies	Cat #1072533
Recombinant DNA		
Plasmid: NK809 dual luciferase	This paper	Addgene #196406
Plasmid: NK810 dual luciferase with *spag6* promoter	This paper	Addgene #196407
Plasmid: NK811 dual luciferase with *spag6* promoter (RFX binding site mutant)	This paper	Addgene #196408
Plasmid: pTH6838 PBM Expression Vector	Timothy R. Hughes	N/A
Software and Algorithms		
MAFFT v. 7.312	Katoh et al 2002^76^, Katoh and Standley 2013^77^	https://mafft.cbrc.jp/alignment/software/
MUSCLE v. 3.8.425	Edgar 2004^85^	https://www.ebi.ac.uk/Tools/msa/muscle/
IQ-TREE v. 2.2.0-beta COVID-edition	Nguyen et al 2015^74^	http://www.iqtree.org/
RAxML v. 8.2.11	Stamatakis 2014^84^	https://cme.hits.org/exelixis/web/software/raxml/
ClipKIT v. 1.3.0	Steenwyk et al 2020^78^	https://github.com/JLSteenwyk/ClipKIT
trimAl v. 1.4.rev22	Capella-Gutiérrez et al 2009^83^	http://trimal.cgenomics.org/
iTOL v. 6	Letunic and Bork 2021^82^	https://itol.embl.de/
PRISM v. 9.0.0	GraphPad	https://www.graphpad.com/
ImageJ v. 2.3.0	ImageJ Software Analysis	https://imagej.net/ij/index.html
Fastqc v. 0.11.9	Babraham Bioinformatics	https://www.bioinformatics.babraham.ac.uk/projects/fastqc/
Salmon v. 1.5.2	Patro et al 2017^88^	https://combine-lab.github.io/salmon/
Trinity v. 2.14.0	Grabherr et al 2011^90^	https://github.com/trinityrnaseq/trinityrnaseq
HOMER v. 4.11	Heinz et al 2010^53^	http://homer.ucsd.edu/homer/
Geneious	Dotmatics	https://www.geneious.com/
Jupyter Notebook	Project Jupyter	https://jupyter.org/
Other		
2100 Bioanalyzer Instrument	Agilent Technologies, QB3 Genomics Lab at UC Berkeley (https://qb3.berkeley.edu/facility/genomics/)	Cat #G2939BA
NovaSeq 6000	Illumina, QB3 Genomics Lab at UC Berkeley	N/A
HiSeq 4000	Illumina, QB3 Genomics Lab at UC Berkeley	N/A
Nucleofector	Lonza	Cat #AAF-1003B, AAG-1003S
Widefield Microscope Axio Observer.Z1/7	Zeiss	N/A
Microscope Objectives: 40X/NA 1.1 LD C-Apochromatic water immersion; 100X/NA 1.40 Plan-Apochromatic oil immersion	Zeiss	N/A
Microscope Camera: Orca-Flash 4.0 LT CMOS Digital Camera	Hamamatsu	N/A
FluoroDish	World Precision Instruments	Cat #FD35–100
SpectraMax M3 plate reader	Molecular Devices	Cat #89429–536
Luna-FL automated cell counter	Logos Biosystems	Cat #L20001

## Highlights

- Choanoflagellates provide a model to reconstruct ciliogenesis regulatory evolution.
- *S. rosetta* has homologs for animal ciliogenic transcription factors RFX and FoxJ1.
- Mutation of cRFXa yields aberrant ciliogenesis and lowers ciliary gene expression.
- *S. rosetta* ciliary gene promoters are enriched for functional RFX binding motifs.

## References

[1] Fritz-Laylin LK (2020). The evolution of animal cell motility. Curr. Biol. 30, R477–R482. 32428485 10.1016/j.cub.2020.03.026

[2] Nielsen C (2008). Six major steps in animal evolution: are we derived sponge larvae? Evol. Dev. 10, 241–257. 18315817 10.1111/j.1525-142X.2008.00231.x

[3] Bloodgood RA (2010). Sensory reception is an attribute of both primary cilia and motile cilia. J. Cell Sci. 123, 505–509. 20144998 10.1242/jcs.066308

[4] Margulis L (1992). Symbiosis in Cell Evolution (W. H. Freeman).

[5] Buss LW (1988). The Evolution of Individuality (Princeton University Press).

[6] Brunet T, and King N (2017). The Origin of Animal Multicellularity and Cell Differentiation. Dev. Cell 43, 124–140. 29065305 10.1016/j.devcel.2017.09.016 PMC6089241

[7] Choksi SP, Lauter G, Swoboda P, and Roy S (2014). Switching on cilia: transcriptional networks regulating ciliogenesis. Development 141, 1427–1441. 24644260 10.1242/dev.074666

[8] Chung M-I, Peyrot SM, LeBoeuf S, Park TJ, McGary KL, Marcotte EM, and Wallingford JB (2012). RFX2 is broadly required for ciliogenesis during vertebrate development. Dev. Biol. 363, 155–165. 22227339 10.1016/j.ydbio.2011.12.029 PMC3640985

[9] Yu X, Ng CP, Habacher H, and Roy S (2008). Foxj1 transcription factors are master regulators of the motile ciliogenic program. Nat. Genet. 40, 1445–1453. 19011630 10.1038/ng.263

[10] Piasecki BP, Burghoorn J, and Swoboda P (2010). Regulatory Factor X (RFX)-mediated transcriptional rewiring of ciliary genes in animals. Proc. Natl. Acad. Sci. U. S. A. 107, 12969–12974. 20615967 10.1073/pnas.0914241107 PMC2919930

[11] Chu JSC, Baillie DL, and Chen N (2010). Convergent evolution of RFX transcription factors and ciliary genes predated the origin of metazoans. BMC Evol. Biol. 10, 130. 20441589 10.1186/1471-2148-10-130 PMC2873420

[12] King N (2004). The unicellular ancestry of animal development. Dev. Cell 7, 313–325. 15363407 10.1016/j.devcel.2004.08.010

[13] Leadbeater BSC (2015). The Choanoflagellates (Cambridge University Press).

[14] Carvalho-Santos Z, Azimzadeh J, Pereira-Leal JB, and Bettencourt-Dias M (2011). Evolution: Tracing the origins of centrioles, cilia, and flagella. J. Cell Biol. 194, 165–175. 21788366 10.1083/jcb.201011152 PMC3144413

[15] Pinskey JM, Lagisetty A, Gui L, Phan N, Reetz E, Tavakoli A, Fu G, and Nicastro D (2022). Three-dimensional flagella structures from animals’ closest unicellular relatives, the Choanoflagellates. Elife 11. 10.7554/eLife.78133. PMC9671500 36384644

[16] Efimenko E, Bubb K, Mak HY, Holzman T, Leroux MR, Ruvkun G, Thomas JH, and Swoboda P (2005). Analysis of xbx genes in C. elegans. Development 132, 1923–1934. 15790967 10.1242/dev.01775

[17] Quigley IK, and Kintner C (2017). Rfx2 Stabilizes Foxj1 Binding at Chromatin Loops to Enable Multiciliated Cell Gene Expression. PLoS Genet. 13, e1006538. 28103240 10.1371/journal.pgen.1006538 PMC5245798

[18] Lemeille S, Paschaki M, Baas D, Morlé L, Duteyrat J-L, Ait-Lounis A, Barras E, Soulavie F, Jerber J, Thomas J, (2020). Interplay of RFX transcription factors 1, 2 and 3 in motile ciliogenesis. Nucleic Acids Res. 48, 9019–9036. 32725242 10.1093/nar/gkaa625 PMC7498320

[19] Swoboda P, Adler HT, and Thomas JH (2000). The RFX-type transcription factor DAF-19 regulates sensory neuron cilium formation in C. elegans. Mol. Cell 5, 411–421. 10882127 10.1016/s1097-2765(00)80436-0

[20] Bonnafe E, Touka M, AitLounis A, Baas D, Barras E, Ucla C, Moreau A, Flamant F, Dubruille R, Couble P, (2004). The transcription factor RFX3 directs nodal cilium development and left-right asymmetry specification. Mol. Cell. Biol. 24, 4417–4427. 15121860 10.1128/MCB.24.10.4417-4427.2004 PMC400456

[21] Dubruille R, Laurençon A, Vandaele C, Shishido E, Coulon-Bublex M, Swoboda P, Couble P, Kernan M, and Durand B (2002). Drosophila regulatory factor X is necessary for ciliated sensory neuron differentiation. Development 129, 5487–5498. 12403718 10.1242/dev.00148

[22] Chen Jianchun, Knowles Heather J., Herbert Jennifer L., and Hackett Brian P. (1998). Mutation of the Mouse Hepatocyte Nuclear Factor/Forkhead Homologue 4 Gene Results in an Absence of Cilia and Random Left-Right Asymmetry. J. Clin. Invest. 233, 575–575. 10.1172/JCI4786 PMC509090 9739041

[23] Stubbs JL, Oishi I, Izpisúa Belmonte JC, and Kintner C (2008). The forkhead protein Foxj1 specifies node-like cilia in Xenopus and zebrafish embryos. Nat. Genet. 40, 1454–1460. 19011629 10.1038/ng.267 PMC4648715

[24] Wu SY, and McLeod M (1995). The sak1 gene of Schizosaccharomyces pombe encodes an RFX family DNA-binding protein that positively regulates cyclic AMP-dependent protein kinase-mediated exit from the mitotic cell cycle. Molecular and Cellular Biology 15, 1479–1488. 10.1128/mcb.15.3.1479. 7862141 PMC230372

[25] Bugeja HE, Hynes MJ, and Andrianopoulos A (2010). The RFX protein RfxA is an essential regulator of growth and morphogenesis in Penicillium marneffei. Eukaryot. Cell 9, 578–591. 20118209 10.1128/EC.00226-09 PMC2863407

[26] Huang M, Zhou Z, and Elledge SJ (1998). The DNA replication and damage checkpoint pathways induce transcription by inhibition of the Crt1 repressor. Cell 94, 595–605. 9741624 10.1016/s0092-8674(00)81601-3

[27] Hao B, Clancy CJ, Cheng S, Raman SB, Iczkowski KA, and Nguyen MH (2009). Candida albicans RFX2 encodes a DNA binding protein involved in DNA damage responses, morphogenesis, and virulence. Eukaryot. Cell 8, 627–639. 19252121 10.1128/EC.00246-08 PMC2669197

[28] Richter DJ, Berney C, Strassert JFH, Poh Y-P, Herman EK, Muñoz-Gómez SA, Wideman JG, Burki F, and de Vargas C (2022). EukProt: a database of genome-scale predicted proteins across the diversity of eukaryotes. Peer Community Journal. 10.24072/pcjournal.173.

[29] Nakagawa S, Gisselbrecht SS, Rogers JM, Hartl DL, and Bulyk ML (2013). DNA-binding specificity changes in the evolution of forkhead transcription factors. Proc. Natl. Acad. Sci. U. S. A. 110, 12349–12354. 23836653 10.1073/pnas.1310430110 PMC3725104

[30] Dayel MJ, Alegado RA, Fairclough SR, Levin TC, Nichols SA, McDonald K, and King N (2011). Cell differentiation and morphogenesis in the colony-forming choanoflagellate Salpingoeca rosetta. Dev. Biol. 357, 73–82. 21699890 10.1016/j.ydbio.2011.06.003 PMC3156392

[31] Nguyen H, Koehl MAR, Oakes C, Bustamante G, and Fauci L (2019). Effects of cell morphology and attachment to a surface on the hydrodynamic performance of unicellular choanoflagellates. J. R. Soc. Interface 16, 20180736. 30958167 10.1098/rsif.2018.0736 PMC6364633

[32] Sedykh I, Keller AN, Yoon B, Roberson L, Moskvin OV, and Grinblat Y (2018). Zebrafish Rfx4 controls dorsal and ventral midline formation in the neural tube. Dev. Dyn. 247, 650–659. 29243319 10.1002/dvdy.24613 PMC5854527

[33] Ashique AM, Choe Y, Karlen M, May SR, Phamluong K, Solloway MJ, Ericson J, and Peterson AS (2009). The Rfx4 transcription factor modulates Shh signaling by regional control of ciliogenesis. Sci. Signal. 2, ra70. 19887680 10.1126/scisignal.2000602

[34] Castro W, Chelbi ST, Niogret C, Ramon-Barros C, Welten SPM, Osterheld K, Wang H, Rota G, Morgado L, Vivier E, (2018). The transcription factor Rfx7 limits metabolism of NK cells and promotes their maintenance and immunity. Nat. Immunol. 19, 809–820. 29967452 10.1038/s41590-018-0144-9 PMC6428260

[35] Manojlovic Z, Earwood R, Kato A, Stefanovic B, and Kato Y (2014). RFX7 is required for the formation of cilia in the neural tube. Mech. Dev. 132, 28–37. 24530844 10.1016/j.mod.2014.02.001 PMC3976564

[36] Booth DS, and King N (2020). Genome editing enables reverse genetics of multicellular development in the choanoflagellate Salpingoeca rosetta. Elife 9, e56193. 32496191 10.7554/eLife.56193 PMC7314544

[37] Brokaw CJ (1960). Decreased adenosine triphosphatase acivity of flagella from a paralyzed mutant of Chlamydomonas moewusii. Exp. Cell Res. 19, 430–432. 13804702 10.1016/0014-4827(60)90027-6

[38] Kistler WS, Baas D, Lemeille S, Paschaki M, Seguin-Estevez Q, Barras E, Ma W, Duteyrat J-L, Morlé L, Durand B, (2015). RFX2 Is a Major Transcriptional Regulator of Spermiogenesis. PLoS Genet. 11, e1005368. 26162102 10.1371/journal.pgen.1005368 PMC4498915

[39] Chung M-I, Kwon T, Tu F, Brooks ER, Gupta R, Meyer M, Baker JC, Marcotte EM, and Wallingford JB (2014). Coordinated genomic control of ciliogenesis and cell movement by RFX2. Elife 3, e01439. 24424412 10.7554/eLife.01439 PMC3889689

[40] Larkins CE, Aviles GDG, East MP, Kahn RA, and Caspary T (2011). Arl13b regulates ciliogenesis and the dynamic localization of Shh signaling proteins. Mol. Biol. Cell 22, 4694–4703. 21976698 10.1091/mbc.E10-12-0994 PMC3226485

[41] Frikstad K-AM, Molinari E, Thoresen M, Ramsbottom SA, Hughes F, Letteboer SJF, Gilani S, Schink KO, Stokke T, Geimer S, (2019). A CEP104-CSPP1 Complex Is Required for Formation of Primary Cilia Competent in Hedgehog Signaling. Cell Rep. 28, 1907–1922.e6. 31412255 10.1016/j.celrep.2019.07.025 PMC6702141

[42] Pathak N, Austin CA, and Drummond IA (2011). Tubulin tyrosine ligase-like genes ttll3 and ttll6 maintain zebrafish cilia structure and motility. J. Biol. Chem. 286, 11685–11695. 21262966 10.1074/jbc.M110.209817 PMC3064220

[43] Sigg MA, Menchen T, Lee C, Johnson J, Jungnickel MK, Choksi SP, Garcia G 3rd, Busengdal H, Dougherty GW, Pennekamp P, (2017). Evolutionary Proteomics Uncovers Ancient Associations of Cilia with Signaling Pathways. Dev. Cell 43, 744–762.e11. 29257953 10.1016/j.devcel.2017.11.014 PMC5752135

[44] Didon L, Zwick RK, Chao IW, Walters MS, Wang R, Hackett NR, and Crystal RG (2013). RFX3 modulation of FOXJ1 regulation of cilia genes in the human airway epithelium. Respir. Res. 14, 70. 23822649 10.1186/1465-9921-14-70 PMC3710277

[45] El Zein L, Ait-Lounis A, Morlé L, Thomas J, Chhin B, Spassky N, Reith W, and Durand B (2009). RFX3 governs growth and beating efficiency of motile cilia in mouse and controls the expression of genes involved in human ciliopathies. J. Cell Sci. 122, 3180–3189. 19671664 10.1242/jcs.048348

[46] Alten L, Schuster-Gossler K, Beckers A, Groos S, Ulmer B, Hegermann J, Ochs M, and Gossler A (2012). Differential regulation of node formation, nodal ciliogenesis and cilia positioning by Noto and Foxj1. Development 139, 1276–1284. 22357932 10.1242/dev.072728

[47] Gajiwala KS, Chen H, Cornille F, Roques BP, Reith W, Mach B, and Burley SK (2000). Structure of the winged-helix protein hRFX1 reveals a new mode of DNA binding. Nature 403, 916–921. 10706293 10.1038/35002634

[48] Reith W, Herrero-Sanchez C, Kobr M, Silacci P, Berte C, Barras E, Fey S, and Mach B (1990). MHC class II regulatory factor RFX has a novel DNA-binding domain and a functionally independent dimerization domain. Genes Dev. 4, 1528–1540. 2253877 10.1101/gad.4.9.1528

[49] Reith W, Kobr M, Emery P, Durand B, Siegrist CA, and Mach B (1994). Cooperative binding between factors RFX and X2bp to the X and X2 boxes of MHC class II promoters. J. Biol. Chem. 269, 20020–20025. 8051086

[50] Badis G, Chan ET, van Bakel H, Pena-Castillo L, Tillo D, Tsui K, Carlson CD, Gossett AJ, Hasinoff MJ, Warren CL, (2008). A library of yeast transcription factor motifs reveals a widespread function for Rsc3 in targeting nucleosome exclusion at promoters. Mol. Cell 32, 878–887. 19111667 10.1016/j.molcel.2008.11.020 PMC2743730

[51] Jolma A, Yan J, Whitington T, Toivonen J, Nitta KR, Rastas P, Morgunova E, Enge M, Taipale M, Wei G, (2013). DNA-binding specificities of human transcription factors. Cell 152, 327–339. 23332764 10.1016/j.cell.2012.12.009

[52] Emery P, Strubin M, Hofmann K, Bucher P, Mach B, and Reith W (1996). A consensus motif in the RFX DNA binding domain and binding domain mutants with altered specificity. Mol. Cell. Biol. 16, 4486–4494. 8754849 10.1128/mcb.16.8.4486 PMC231447

[53] Heinz S, Benner C, Spann N, Bertolino E, Lin YC, Laslo P, Cheng JX, Murre C, Singh H, and Glass CK (2010). Simple combinations of lineage-determining transcription factors prime cis-regulatory elements required for macrophage and B cell identities. Mol. Cell 38, 576–589. 20513432 10.1016/j.molcel.2010.05.004 PMC2898526

[54] Medina EM, and Buchler NE (2020). Chytrid fungi. Curr. Biol. 30, R516–R520. 32428492 10.1016/j.cub.2020.02.076

[55] Lam KN, van Bakel H, Cote AG, van der Ven A, and Hughes TR (2011). Sequence specificity is obtained from the majority of modular C2H2 zinc-finger arrays. Nucleic Acids Res. 39, 4680–4690. 21321018 10.1093/nar/gkq1303 PMC3113560

[56] Weirauch MT, Cote A, Norel R, Annala M, Zhao Y, Riley TR, Saez-Rodriguez J, Cokelaer T, Vedenko A, Talukder S, (2013). Evaluation of methods for modeling transcription factor sequence specificity. Nat. Biotechnol. 31, 126–134. 23354101 10.1038/nbt.2486 PMC3687085

[57] Weirauch MT, Yang A, Albu M, Cote AG, Montenegro-Montero A, Drewe P, Najafabadi HS, Lambert SA, Mann I, Cook K, (2014). Determination and inference of eukaryotic transcription factor sequence specificity. Cell 158, 1431–1443. 25215497 10.1016/j.cell.2014.08.009 PMC4163041

[58] Sugiaman-Trapman D, Vitezic M, Jouhilahti E-M, Mathelier A, Lauter G, Misra S, Daub CO, Kere J, and Swoboda P (2018). Characterization of the human RFX transcription factor family by regulatory and target gene analysis. BMC Genomics 19, 181. 29510665 10.1186/s12864-018-4564-6 PMC5838959

[59] Booth DS, Szmidt-Middleton H, and King N (2018). Transfection of choanoflagellates illuminates their cell biology and the ancestry of animal septins. Mol. Biol. Cell 29, 3026–3038. 30281390 10.1091/mbc.E18-08-0514 PMC6333174

[60] Richter DJ, Fozouni P, Eisen MB, and King N (2018). Gene family innovation, conservation and loss on the animal stem lineage. Elife 7. 10.7554/eLife.34226. PMC6040629 29848444

[61] Dayel MJ, and King N (2014). Prey capture and phagocytosis in the choanoflagellate Salpingoeca rosetta. PLoS One 9, e95577. 24806026 10.1371/journal.pone.0095577 PMC4012994

[62] Mackie GO (1970). Neuroid conduction and the evolution of conducting tissues. Q. Rev. Biol. 45, 319–332. 4395914 10.1086/406645

[63] Arendt D (2008). The evolution of cell types in animals: emerging principles from molecular studies. Nat. Rev. Genet. 9, 868–882. 18927580 10.1038/nrg2416

[64] Mikhailov KV, Konstantinova AV, Nikitin MA, Troshin PV, Rusin LY, Lyubetsky VA, Panchin YV, Mylnikov AP, Moroz LL, Kumar S, (2009). The origin of Metazoa: a transition from temporal to spatial cell differentiation. Bioessays 31, 758–768. 19472368 10.1002/bies.200800214

[65] Zakhvatkin AA (1949). The comparative embryology of the low invertebrates. Sources and method of the origin of metazoan development. Soviet Science.

[66] Wagner GP, Erkenbrack EM, and Love AC (2019). Stress-Induced Evolutionary Innovation: A Mechanism for the Origin of Cell Types. Bioessays 41, e1800188. 30919472 10.1002/bies.201800188 PMC7202399

[67] Kin K, Chen Z-H, Forbes G, and Schaap P (2022). Evolution of a novel cell type in Dictyostelia required gene duplication of a cudA-like transcription factor. Curr. Biol. 32, 428–437.e4. 34883046 10.1016/j.cub.2021.11.047 PMC8808424

[68] Ohno S (1970). Evolution by Gene Duplication (Springer Science & Business Media).

[69] Adl SM, Simpson AGB, Lane CE, Lukeš J, Bass D, Bowser SS, Brown MW, Burki F, Dunthorn M, Hampl V, (2012). The revised classification of eukaryotes. J. Eukaryot. Microbiol. 59, 429–493. 23020233 10.1111/j.1550-7408.2012.00644.x PMC3483872

[70] Dunn CW, Hejnol A, Matus DQ, Pang K, Browne WE, Smith SA, Seaver E, Rouse GW, Obst M, Edgecombe GD, (2008). Broad phylogenomic sampling improves resolution of the animal tree of life. Nature 452, 745–749. 18322464 10.1038/nature06614

[71] Philippe H, Brinkmann H, Lavrov DV, Littlewood DTJ, Manuel M, Wörheide G, and Baurain D (2011). Resolving difficult phylogenetic questions: why more sequences are not enough. PLoS Biol. 9, e1000602. 21423652 10.1371/journal.pbio.1000602 PMC3057953

[72] King N, and Rokas A (2017). Embracing Uncertainty in Reconstructing Early Animal Evolution. Curr. Biol. 27, R1081–R1088. 29017048 10.1016/j.cub.2017.08.054 PMC5679448

[73] Carr M, Richter DJ, Fozouni P, Smith TJ, Jeuck A, Leadbeater BSC, and Nitsche F (2017). A six-gene phylogeny provides new insights into choanoflagellate evolution. Mol. Phylogenet. Evol. 107, 166–178. 27765632 10.1016/j.ympev.2016.10.011

[74] Nguyen L-T, Schmidt HA, von Haeseler A, and Minh BQ (2015). IQ-TREE: a fast and effective stochastic algorithm for estimating maximum-likelihood phylogenies. Mol. Biol. Evol. 32, 268–274. 25371430 10.1093/molbev/msu300 PMC4271533

[75] Vij S, Rink JC, Ho HK, Babu D, Eitel M, Narasimhan V, Tiku V, Westbrook J, Schierwater B, and Roy S (2012). Evolutionarily ancient association of the FoxJ1 transcription factor with the motile ciliogenic program. PLoS Genet. 8, e1003019. 23144623 10.1371/journal.pgen.1003019 PMC3493443

[76] Katoh K, Misawa K, Kuma K-I, and Miyata T (2002). MAFFT: a novel method for rapid multiple sequence alignment based on fast Fourier transform. Nucleic Acids Res. 30, 3059–3066. 12136088 10.1093/nar/gkf436 PMC135756

[77] Katoh K, and Standley DM (2013). MAFFT multiple sequence alignment software version 7: improvements in performance and usability. Mol. Biol. Evol. 30, 772–780. 23329690 10.1093/molbev/mst010 PMC3603318

[78] Steenwyk JL, Buida TJ 3rd, Li Y, Shen X-X, and Rokas A (2020). ClipKIT: A multiple sequence alignment trimming software for accurate phylogenomic inference. PLoS Biol. 18, e3001007. 33264284 10.1371/journal.pbio.3001007 PMC7735675

[79] Kalyaanamoorthy S, Minh BQ, Wong TKF, von Haeseler A, and Jermiin LS (2017). ModelFinder: fast model selection for accurate phylogenetic estimates. Nat. Methods 14, 587–589. 28481363 10.1038/nmeth.4285 PMC5453245

[80] Minh BQ, Nguyen MAT, and von Haeseler A (2013). Ultrafast approximation for phylogenetic bootstrap. Mol. Biol. Evol. 30, 1188–1195. 23418397 10.1093/molbev/mst024 PMC3670741

[81] Guindon S, Dufayard J-F, Lefort V, Anisimova M, Hordijk W, and Gascuel O (2010). New algorithms and methods to estimate maximum-likelihood phylogenies: assessing the performance of PhyML 3.0. Syst. Biol. 59, 307–321. 20525638 10.1093/sysbio/syq010

[82] Letunic I, and Bork P (2021). Interactive Tree Of Life (iTOL) v5: an online tool for phylogenetic tree display and annotation. Nucleic Acids Res. 49, W293–W296. 33885785 10.1093/nar/gkab301 PMC8265157

[83] Capella-Gutiérrez S, Silla-Martínez JM, and Gabaldón T (2009). trimAl: a tool for automated alignment trimming in large-scale phylogenetic analyses. Bioinformatics 25, 1972–1973. 19505945 10.1093/bioinformatics/btp348 PMC2712344

[84] Stamatakis A (2014). RAxML version 8: a tool for phylogenetic analysis and post-analysis of large phylogenies. Bioinformatics 30, 1312–1313. 24451623 10.1093/bioinformatics/btu033 PMC3998144

[85] López-Escardó D, Grau-Bové X, Guillaumet-Adkins A, Gut M, Sieracki ME, and Ruiz-Trillo I (2019). Reconstruction of protein domain evolution using single-cell amplified genomes of uncultured choanoflagellates sheds light on the origin of animals. Philos. Trans. R. Soc. Lond. B Biol. Sci. 374, 20190088. 31587642 10.1098/rstb.2019.0088 PMC6792448

[86] Needham DM, Yoshizawa S, Hosaka T, Poirier C, Choi CJ, Hehenberger E, Irwin NAT, Wilken S, Yung C-M, Bachy C, (2019). A distinct lineage of giant viruses brings a rhodopsin photosystem to unicellular marine predators. Proc. Natl. Acad. Sci. U. S. A. 116, 20574–20583. 31548428 10.1073/pnas.1907517116 PMC6789865

[87] Manni M, Berkeley MR, Seppey M, Simão FA, and Zdobnov EM (2021). BUSCO Update: Novel and Streamlined Workflows along with Broader and Deeper Phylogenetic Coverage for Scoring of Eukaryotic, Prokaryotic, and Viral Genomes. Mol. Biol. Evol. 38, 4647–4654. 34320186 10.1093/molbev/msab199 PMC8476166

[88] Edgar RC (2004). MUSCLE: multiple sequence alignment with high accuracy and high throughput. Nucleic Acids Res. 32, 1792–1797. 15034147 10.1093/nar/gkh340 PMC390337

[89] Alegado RA, Brown LW, Cao S, Dermenjian RK, Zuzow R, Fairclough SR, Clardy J, and King N (2012). A bacterial sulfonolipid triggers multicellular development in the closest living relatives of animals. Elife 1, e00013. 23066504 10.7554/eLife.00013 PMC3463246

[90] Fairclough SR, Chen Z, Kramer E, Zeng Q, Young S, Robertson HM, Begovic E, Richter DJ, Russ C, Westbrook MJ, (2013). Premetazoan genome evolution and the regulation of cell differentiation in the choanoflagellate Salpingoeca rosetta. Genome Biol. 14, R15. 23419129 10.1186/gb-2013-14-2-r15 PMC4054682

[91] Patro R, Duggal G, Love MI, Irizarry RA, and Kingsford C (2017). Salmon provides fast and bias-aware quantification of transcript expression. Nat. Methods 14, 417–419. 28263959 10.1038/nmeth.4197 PMC5600148

[92] Robinson MD, McCarthy DJ, and Smyth GK (2010). edgeR: a Bioconductor package for differential expression analysis of digital gene expression data. Bioinformatics 26, 139–140. 19910308 10.1093/bioinformatics/btp616 PMC2796818

[93] Grabherr MG, Haas BJ, Yassour M, Levin JZ, Thompson DA, Amit I, Adiconis X, Fan L, Raychowdhury R, Zeng Q, (2011). Full-length transcriptome assembly from RNA-Seq data without a reference genome. Nat. Biotechnol. 29, 644–652. 21572440 10.1038/nbt.1883 PMC3571712

[94] Schindelin J, Arganda-Carreras I, Frise E, Kaynig V, Longair M, Pietzsch T, Preibisch S, Rueden C, Saalfeld S, Schmid B, (2012). Fiji: an open-source platform for biological-image analysis. Nat. Methods 9, 676–682. 22743772 10.1038/nmeth.2019 PMC3855844

[95] Gherman Adrian, Davis Erica E., and Katsanis Nicholas (2006). The ciliary proteome database: an integrated community resource for the genetic and functional dissection of cilia. Nat. Genet. 10.1038/ng0906-961 16940995

[96] Arnaiz O, Malinowska A, Klotz C, Sperling L, Dadlez M, Koll F, and Cohen J (2009). Cildb: a knowledgebase for centrosomes and cilia. Database 2009, bap022. 10.1093/database/bap022 PMC2860946 20428338

[97] van Dam TJ, Wheway G, Slaats GG, SYSCILIA Study Group, Huynen MA., and Giles RH. (2013). The SYSCILIA gold standard (SCGSv1) of known ciliary components and its applications within a systems biology consortium. Cilia 2, 7. 23725226 10.1186/2046-2530-2-7 PMC3674929

[98] Jones P, Binns D, Chang H-Y, Fraser M, Li W, McAnulla C, McWilliam H, Maslen J, Mitchell A, Nuka G, (2014). InterProScan 5: genome-scale protein function classification. Bioinformatics 30, 1236–1240. 24451626 10.1093/bioinformatics/btu031 PMC3998142

