# Supplementary material for: An RFX transcription factor regulates ciliogenesis in the closest living relatives of animals
Source: Curr Biol. Author manuscript; Available in PMC 2023 Sep 27. (PMC10530576; doi:10.1016/j.cub.2023.07.022)
Supplement: Supplementary Figures [file NIHMS1923668-supplement-Supplementary_Figures.pdf]

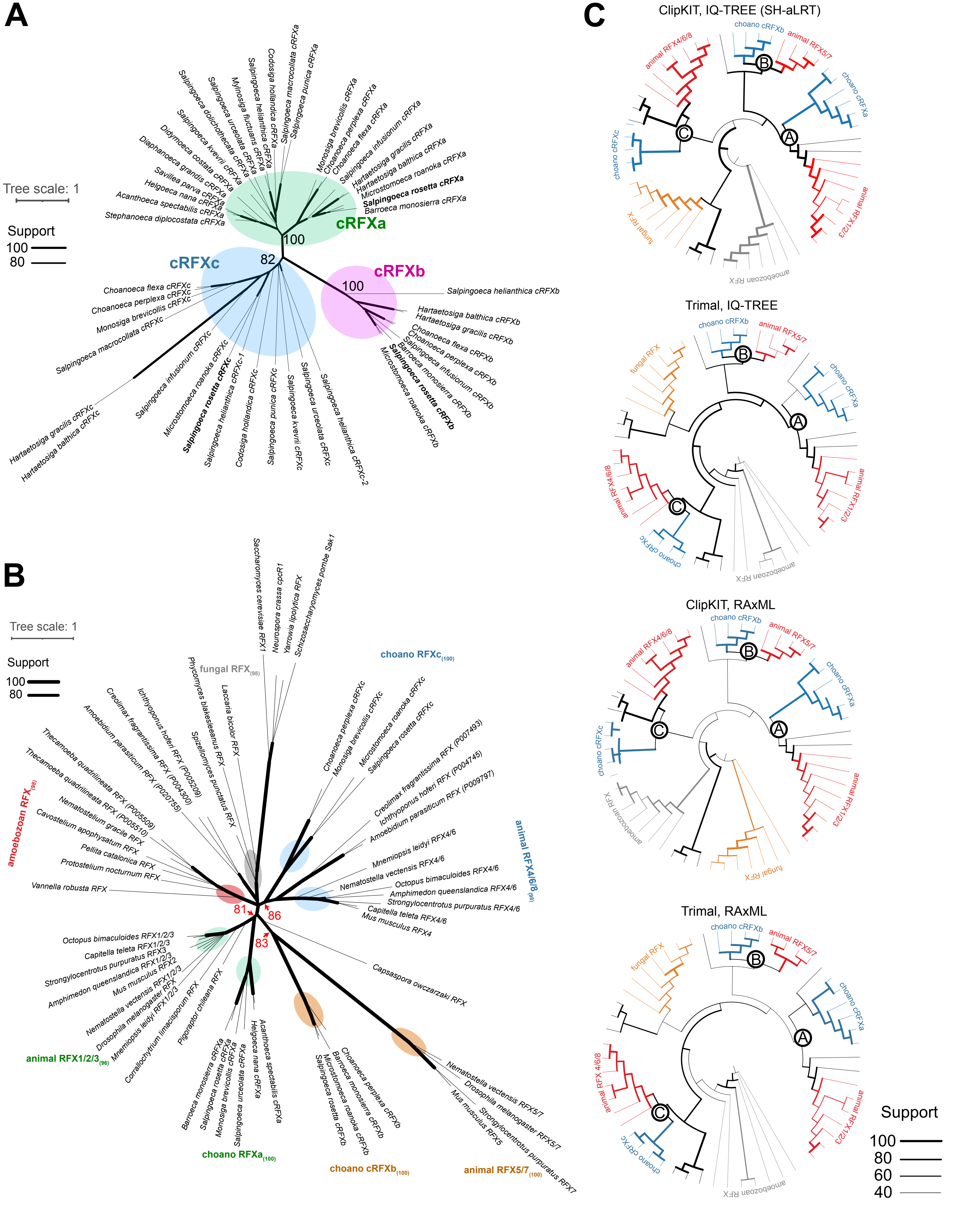

A
Tree scale: 1
Support
100
80
cRFXa
cRFXb
cRFXc
100
82
100
Codosiga hollandica cRFXa
Salpingoeca macrocollata cRFXa
Salpingoeca punica cRFXa
Salpingoeca helianthica cRFXa
Mylnosiga fluctuans cRFXa
Salpingoeca urceolata cRFXa
Salpingoeca dolichothecata cRFXa
Salpingoeca kvevrii cRFXa
Didymoeca costata cRFXa
Diaphanoeca grandis cRFXa
Savillea parva cRFXa
Helgoeca nana cRFXa
Acanthoeca spectabilis cRFXa
Stephanoeca diplocostata cRFXa
Monosiga brevicollis cRFXa
Choanoeca perplexa cRFXa
Choanoeca flexa cRFXa
Salpingoeca infusionum cRFXa
Hartaetosiga gracilis cRFXa
Hartaetosiga balthica cRFXa
Microstomoeca roanoka cRFXa
Salpingoeca rosetta cRFXa
Barroeca monosierra cRFXa
Salpingoeca helianthica cRFXb
Hartaetosiga balthica cRFXb
Hartaetosiga gracilis cRFXb
Choanoeca flexa cRFXb
Choanoeca perplexa cRFXb
Salpingoeca infusionum cRFXb
Barroeca monosierra cRFXb
Salpingoeca rosetta cRFXb
Microstomoeca roanoka cRFXb
Choanoeca flexa cRFXc
Choanoeca perplexa cRFXc
Monosiga brevicollis cRFXc
Salpingoeca macrocollata cRFXc
Salpingoeca infusionum cRFXc
Microstomoeca roanoka cRFXc
Salpingoeca rosetta cRFXc
Salpingoeca helianthica cRFXc-1
Codosiga hollandica cRFXc
Salpingoeca punica cRFXc
Salpingoeca kvevrii cRFXc
Salpingoeca urceolata cRFXc
Salpingoeca helianthica cRFXc-2
Hartaetosiga gracilis cRFXc
Hartaetosiga balthica cRFXc
B
Tree scale: 1
Support
100
80
Saccharomyces cerevisiae RFX1
Neurospora crassa cpcR1
Yarrowia lipolytica RFX
Schizosaccharyomyces pombe Sak1
fungal RFX(98)
Phycomyces blakesleeanus RFX
Laccaria bicolor RFX
Spizellomyces punctatus RFX
choano RFXc(100)
Choanoeca perplexa cRFXc
Monosiga brevicollis cRFXc
Microstomoeca roanoka cRFXc
Salpingoeca rosetta cRFXc
Creolimax fragrantissima RFX (P007493)
Ichthyophonus hoferi RFX (P004745)
Amoebidium parasiticum RFX (P009797)
Creolimax fragrantissima RFX (P005209)
Ichthyophonus hoferi RFX (P004300)
Amoebidium parasiticum RFX (P020755)
Thecamoeba quadrilineata RFX (P005509)
Thecamoeba quadrilineata RFX (P005510)
Nematostelium gracile RFX
Cavostelium apophysatum RFX
Pellita catalonica RFX
Protostelium nocturnum RFX
Vannella robusta RFX
amoebozoan RFX(98)
animal RFX4/6/8(98)
Mnemiopsis leidyi RFX4/6
Nematostella vectensis RFX4/6
Octopus bimaculoides RFX4/6
Amphimedon queenslandica RFX4/6
Strongylocentrotus purpuratus RFX4/6
Capitella teleta RFX4/6
Mus musculus RFX4
81
86
83
Capsaspora owczarzaki RFX
Octopus bimaculoides RFX1/2/3
Capitella teleta RFX1/2/3
Strongylocentrotus purpuratus RFX3
Amphimedon queenslandica RFX1/2/3
Mus musculus RFX2
Nematostella vectensis RFX1/2/3
Drosophila melanogaster RFX
Mnemiopsis leidyi RFX1/2/3
Corallochytrium limacisporum RFX
Pigoraptor chileana RFX
animal RFX1/2/3(96)
Barroeca monosierra cRFXa
Salpingoeca rosetta cRFXa
Monosiga brevicollis cRFXa
Salpingoeca urceolata cRFXa
Helgoeca nana cRFXa
Acanthoeca spectabilis cRFXa
choano RFXa(100)
Choanoeca perplexa cRFXb
Barroeca monosierra cRFXb
Microstomoeca roanoka cRFXb
Salpingoeca rosetta cRFXb
choano cRFXb(100)
Nematostella vectensis RFX5/7
Drosophila melanogaster RFX5/7
Mus musculus RFX5
Strongylocentrotus purpuratus RFX7
animal RFX5/7(100)
C
ClipKIT, IQ-TREE (SH-aLRT)
choano cRFXb
animal RFX5/7
animal RFX4/6/8
choano cRFXa
choano cRFXc
animal RFX1/2/3
fungal RFX
amoebozoan RFX
A
B
C
Trimal, IQ-TREE
choano cRFXb
animal RFX5/7
fungal RFX
choano cRFXa
animal RFX4/6/8
animal RFX1/2/3
choano cRFXc
amoebozoan RFX
A
B
C
ClipKIT, RAxML
choano cRFXb
animal RFX5/7
animal RFX4/6/8
choano cRFXa
choano cRFXc
animal RFX1/2/3
amoebozoan RFX
fungal RFX
A
B
C
Trimal, RAxML
choano cRFXb
animal RFX5/7
fungal RFX
choano cRFXa
animal RFX 4/6/8
animal RFX1/2/3
choano cRFXc
amoebozoan RFX
A
B
C
Support
100
80
60
40

Figure S1. Phylogenetic trees of RFX genes. Related to Figure 1.

(A) Choanoflagellate RFX genes form three sub-families. Choanoflagellate RFX protein sequences (File S1) were aligned with MAFFT, trimmed with ClipKIT, and assembled into a maximum-likelihood phylogenetic tree with IQ-TREE. Width of branches indicate UF-boot support. All nodes with less than 75% bootstrap support are collapsed. Every choanoflagellate with RFX genes contains a copy of *cRFXa* (green, 100% UF-boot support), while *cRFXb* (pink, 100% UF-boot support) and *cRFXc* (blue, 82% UF-boot support) are found in subsets of choanoflagellate taxa. Tree scale indicates length of branch corresponding to one substitution per site in amino acid alignment.

(B) Choanoflagellate *cRFXa* genes are orthologous to the animal *RFX1/2/3* sub-family, *cRFXb* genes are orthologous to animal *RFX5/7*, and *cRFXc* genes are orthologous to animal *RFX4/6/8*. Selected RFX protein sequences from across diverse opisthokonts and amoebozoans (File S1) were aligned with MAFFT, trimmed with ClipKIT, and assembled into a maximum-likelihood phylogenetic tree with IQ-TREE. The three previously discovered choanoflagellate RFX families were well-resolved, as were the three animal RFX families, fungal RFX genes, and amoebozoan RFX genes. Red letters and arrows indicate UF-boot support for nodes that connect animal and choanoflagellate RFX gene families. Note that ichthyosporeans (*A. parasiticum*, *C. fragrantissima*, *I. hoferi*) contain at least two RFX genes, one of which groups with *cRFXc* and *aRFX4/6/8*. Width of branches indicates bootstrap support and all nodes with less than 75% bootstrap support are collapsed. Tree scale indicates length of branch corresponding to one substitution per site in amino acid alignment.

(C) Different phylogenetic software packages recover similar phylogenetic relationships between animal and choanoflagellate RFX sub-families. A set of opisthokont and amoebozoan full-length RFX protein sequences (File S1, the same sequences used for Figure 1D and Figure S1B) were aligned with MAFFT, followed by alignment trimming with either ClipKIT or trimAl, and then maximum-likelihood tree construction with either IQ-TREE or RAxML. For both ML

algorithms, automatic best model finding was used. For the combination of ClipKIT and IQ-TREE, we show SH-aLRT statistics (1000 iterations), for comparison with UF-boot statistics (the tree shown in Figure 1D). For Trimal/IQ-TREE, 1000 Ultrafast bootstraps were used. For RAxML trees, 100 bootstraps were used. Width of branches indicates bootstrap support. Animal, choanoflagellate, fungal, and amoebozoan RFX sub-families are indicated. Labels A,B,C indicate ancestral nodes of homologous choanoflagellate/animal RFX sub-families. Bootstrap supports are: ClipKIT/IQ-TREE/SH-aLRT (A: 66%, B: 89%, C: 58%); Trimal/IQ-TREE (A: 72%, B: 78%, C: 96%); ClipKIT/RAxML/Bootstrap (A: 19%, B: 39%, C: 30%); Trimal/RAxML/Bootstrap (A: 26%, B: 29%, C: 71%).

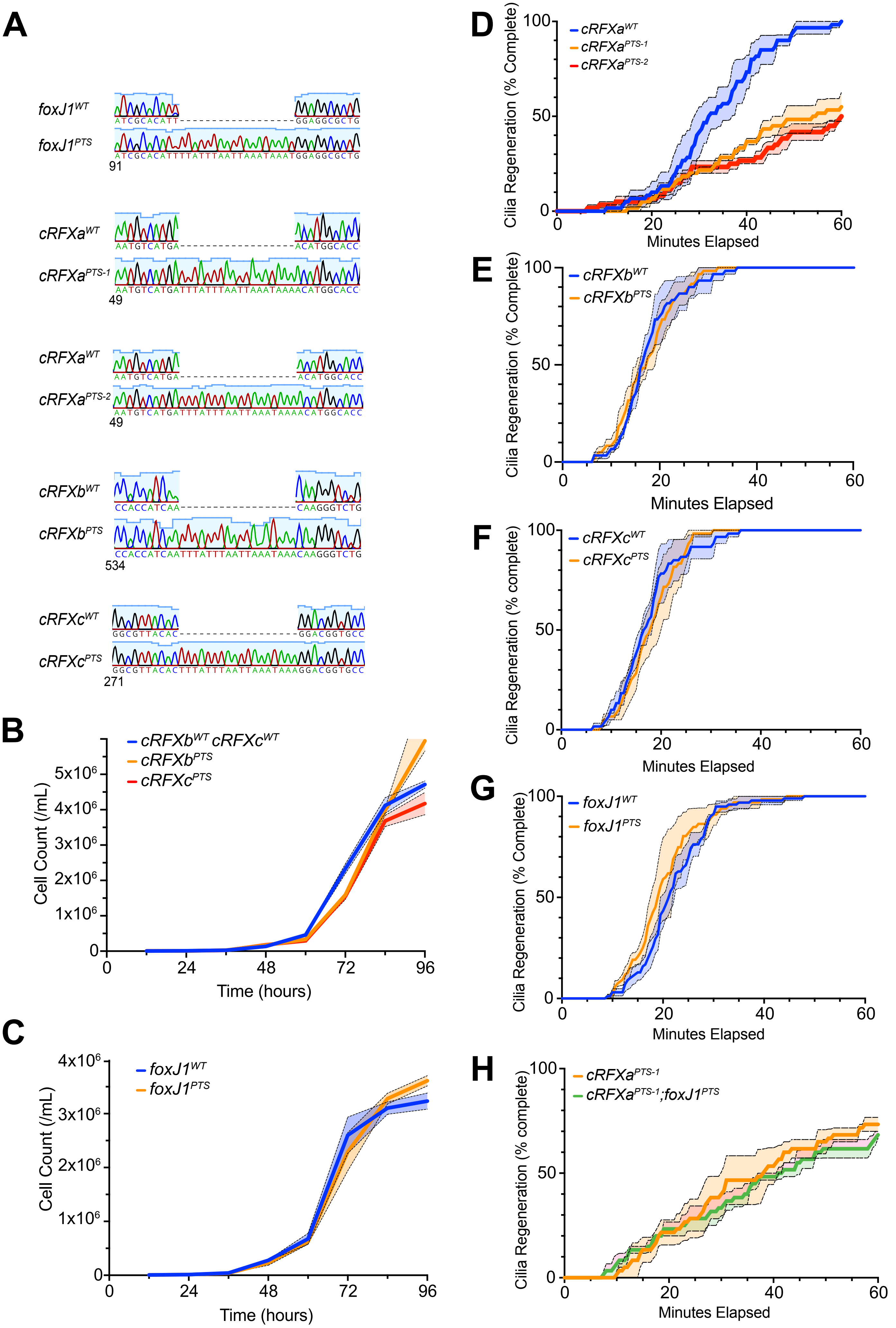

A
foxJ1WT
ATCGCACATT
GGAGGCGCTG
foxJ1PTS
ATCGCACATTTTATTTAATTAAATAAATGGAGGCGCTG
91
cRFXaWT
AATGTCATGA
ACATGGCACC
cRFXaPTS-1
AATGTCATGATTTATTTAATTAAATAAAACATGGCACC
49
cRFXaWT
AATGTCATGA
ACATGGCACC
cRFXaPTS-2
AATGTCATGATTTATTTAATTAAATAAAACATGGCACC
49
cRFXbWT
CCACCATCAA
CAAGGGTCTG
cRFXbPTS
CCACCATCAATTTATTTAATTAAATAAACAAGGGTCTG
534
cRFXcWT
GGCGTTACAC
GGACGGTGCC
cRFXcPTS
GGCGTTACACTTTATTTAATTAAATAAAGGACGGTGCC
271
B
cRFXbWT cRFXcWT
cRFXbPTS
cRFXcPTS
Cell Count (/mL)
Time (hours)
C
foxJ1WT
foxJ1PTS
Cell Count (/mL)
Time (hours)
D
cRFXaWT
cRFXaPTS-1
cRFXaPTS-2
Cilia Regeneration (% Complete)
Minutes Elapsed
E
cRFXbWT
cRFXbPTS
Cilia Regeneration (% Complete)
Minutes Elapsed
F
cRFXcWT
cRFXcPTS
Cilia Regeneration (% complete)
Minutes Elapsed
G
foxJ1WT
foxJ1PTS
Cilia Regeneration (% Complete)
Minutes Elapsed
H
cRFXaPTS-1
cRFXaPTS-1;foxJ1PTS
Cilia Regeneration (% complete)
Minutes Elapsed

Figure S2. Genotyping, growth rates, and ciliogenesis of transcription factor mutant strains. Related to Figure 2.

(A) Clonally isolated cells from CRISPR genome editing experiments were genotyped by PCR and Sanger sequencing (File S3). Numbers show relative position in the coding DNA sequence of the target gene. The TTTATTTAATTAAATAAA cassette is introduced in an exon and creates a stop codon in every possible reading frame. All genes are truncated before the DNA-binding domain.

(B) *cRFXb*^{PTS} and *cRFXc*^{PTS} strains show equivalent proliferation rates compared to an isogenic strain (Materials and Methods). As in Figure 2B, cells were diluted to 1,000 cells / ml and triplicate samples were collected and counted every 12 hours for 96 hours. The mean values are plotted with the standard error of the mean shown as dotted lines.

(C) *foxJ1*^{PTS} shows an equivalent proliferation rate compared to an isogenic strain. Growth rates were assayed and quantified as in Figure 2B.

(D) Ciliogenesis for *cRFXa*^{WT}, *cRFXa*$^{PTS\text{-}1}$, and *cRFXa*$^{PTS\text{-}2}$ was compared under standard growth conditions as described in Figure 2G. For each strain, triplicate experiments were done, quantifying the time point of completed regeneration for each of 20 cells, and plotting the percent that have completed ciliary regeneration as a function of time. Dotted lines show standard error of the mean across the three replicates.

(E) The *cRFXb*^{PTS} strain shows no defect in ciliogenesis. The data represents the average of three triplicate experiments (n=20 cells each) plotting the percent that have completed ciliary regeneration as a function of time. Dotted lines represent standard error of the mean.

(F) The *cRFXc*^{PTS} strain shows no defect in ciliogenesis. The data represents the average of three triplicate experiments (n=20 cells each) plotting the percent that have completed ciliary regeneration as a function of time. Dotted lines represent standard error of the mean.

(G) The *foxJ1*^{PTS} strain shows no defect in ciliogenesis. The data represents the average of three triplicate experiments (n=20+ cells each) plotting the percent that have completed ciliary regeneration as a function of time. Dotted lines represent standard error of the mean.

(H) The *cRFXa*^{PTS}*foxJ1*^{PTS} double mutant strain shows a ciliogenesis defect comparable to that observed in *cRFXa*$^{PTS\text{-}1}$. The data represents the average of three triplicate experiments (n=20 cells each) plotting the percent that have completed ciliary regeneration as a function of time. Dotted lines represent standard error of the mean.

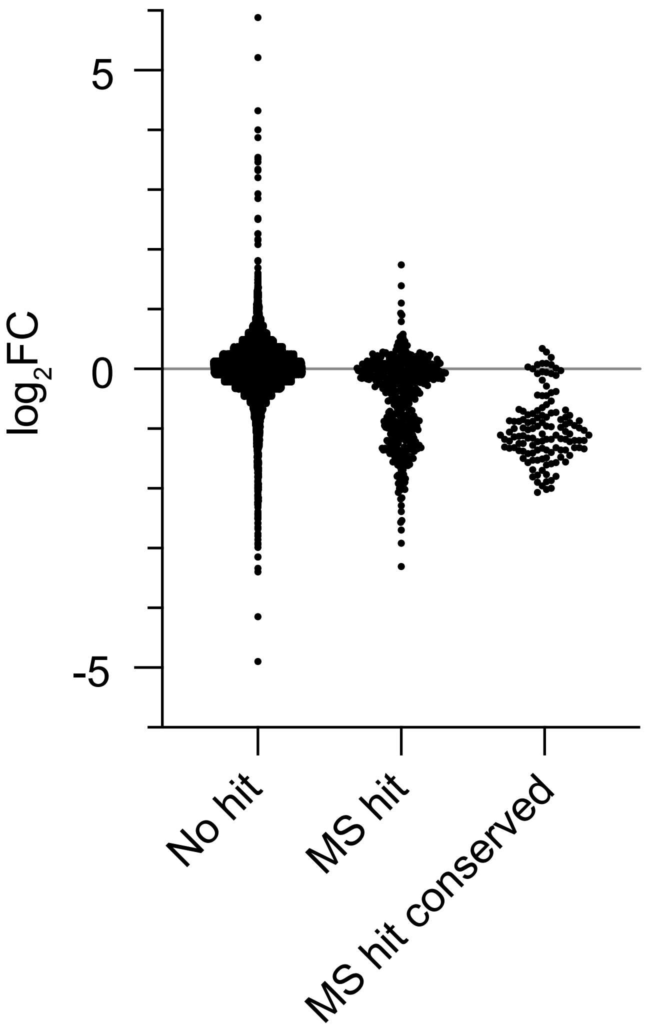

5
0
-5
log2FC
No hit
MS hit
MS hit conserved

Figure S3. Genes with protein products identified in *S. rosetta* cilia by mass spectrometry (MS) are down-regulated in *cRFXa*$^{PTS\text{-}1}$ cells. Related to Figure 3.

Dots show $\log_2$FC values for proteins not identified by MS in the cilia, genes that were identified by MS in cilia, and a subset of the MS hits: proteins whose sea urchin and sea anemone orthologs were also identified in the ciliary proteome of those respective taxa (MS hit conserved). MS data from Sigg *et al,* with 464 proteins identified in the *S. rosetta* ciliome. 131 of these are likely to have conserved ciliary function across Choanozoa, due to the presence of orthologs detected in the ciliary proteomes of sea urchins and sea anemones. Transcripts whose products were detected in the ciliary proteome were on average down-regulated in *cRFXa*$^{PTS\text{-}1}$ mutant cells (avg $\log_2$FC = -0.50), and the subset of choanozoan-conserved ciliary genes showed more extensive down-regulation ($\log_2$FC = -1.00), suggesting that ciliary genes with evolutionarily conserved function have greater dependence on RFX-mediated transcriptional regulation in *S. rosetta*.

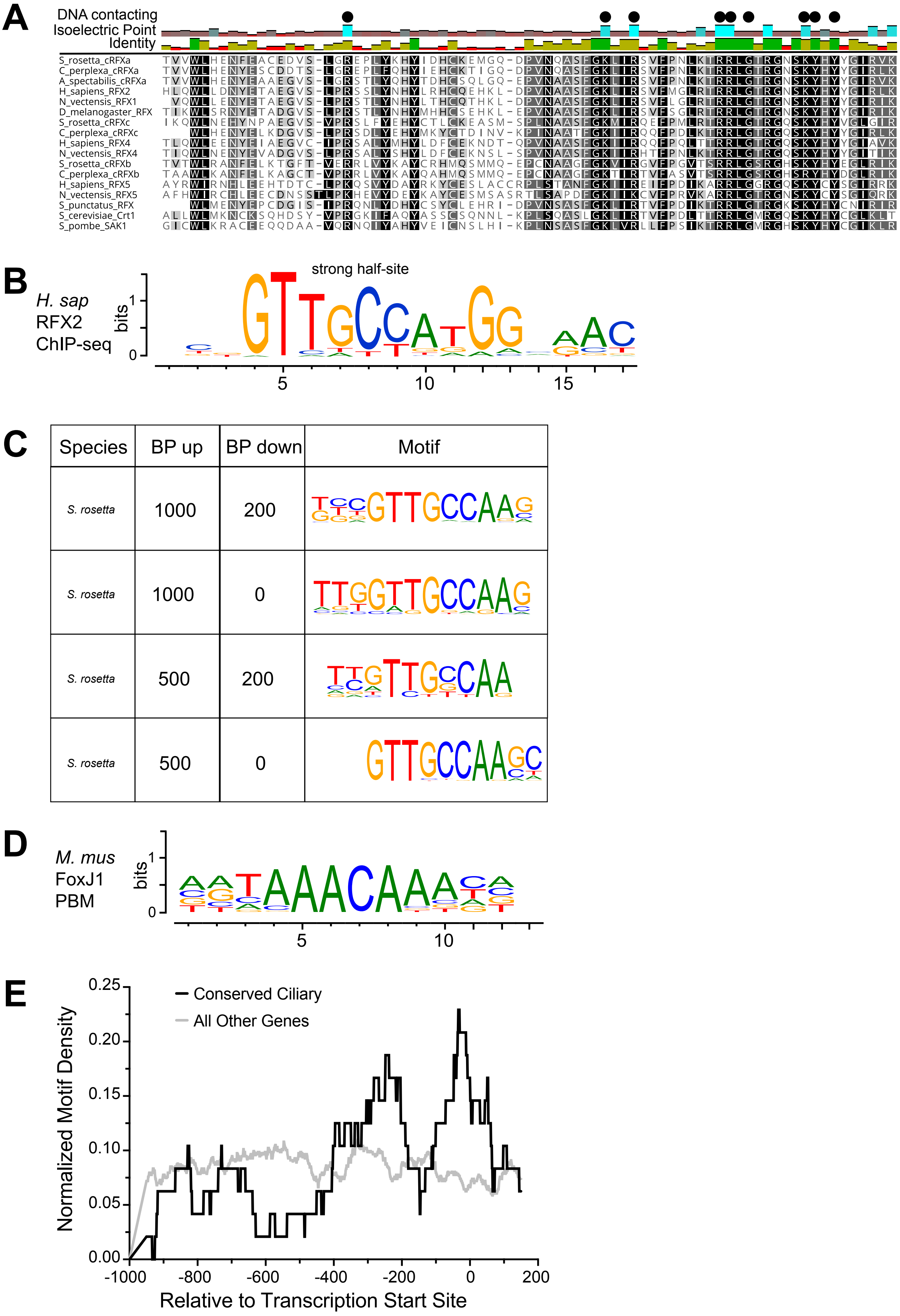
A
DNA contacting
Isoelectric Point
Identity
S_rosetta_cRFXa
C_perplexa_cRFXa
A_spectabilis_cRFXa
H_sapiens_RFX2
N_vectensis_RFX1
D_melanogaster_RFX
S_rosetta_cRFXc
C_perplexa_cRFXc
H_sapiens_RFX4
N_vectensis_RFX4
S_rosetta_cRFXb
C_perplexa_cRFXb
H_sapiens_RFX5
N_vectensis_RFX5
S_punctatus_RFX
S_cerevisiae_Crt1
S_pombe_SAK1
B
H. sap
RFX2
ChIP-seq
strong half-site
bits
C
Species
BP up
BP down
Motif
S. rosetta
1000
200
S. rosetta
1000
0
S. rosetta
500
200
S. rosetta
500
0
D
M. mus
FoxJ1
PBM
bits
E
Conserved Ciliary
All Other Genes
Normalized Motif Density
Relative to Transcription Start Site

Figure S4. DNA-binding motifs for RFX and FoxJ1 transcription factors. Related to Figure 4.

(A) RFX DNA-binding domain sequences have highly conserved DNA contacting residues. Selected RFX DNA-binding domains were aligned with MUSCLE and individual residues shaded according to identity. DNA contacting residues as determined by a crystal structure of *H. sapiens* RFX1 are labeled with black circles. These largely basic residues (note their correspondence with the average isoelectric point of each residue in the alignment) are almost perfectly conserved across all RFX sequences.

(B) The *H. sapiens* RFX2 consensus motif derived from ChIP-seq (JASPAR MA0600.1). This motif consists of two inverted, palindromic half-sites, one of which (here shown on the left) has stricter specificity requirements. The DNA binding specificity for RFX TFs is conserved across animal RFX proteins[10,16,17].

(C) Identification of a ciliome-enriched RFX motif is robust to definitions of promoter length. RFX-like motifs are identified as the most enriched in *S. rosetta* ciliome promoters across different definitions of promoter length, relative to annotated transcription start sites. Promoters were extracted using the criteria displayed and analyzed for motif enrichment using HOMER and our set of HsaSro conserved ciliary genes (File S6).

(D) The DNA binding preferences of *Mus musculus* FoxJ1 as determined by PBM (Cis-BP ID #M00161_2.00). The consensus motif is distinct from RFX binding motifs and does not show similarity to the motif identified as enriched in HsaSro ciliary promoter regions.

(E) *M. brevicollis* RFX motifs are preferentially located near transcription start sites of ciliome genes. The motif density within promoters is shown for motif instances in conserved ciliome genes, as well as motif instances in all other promoters. The RFX motif identified by HOMER (Figure 4B) in *M. brevicollis* was used. Normalized motif density (y-axis) describes the proportion of all motifs that fall into a 100 bp sliding window centered on any given position on the x-axis. The x-

axis gives promoter position relative to the predicted transcription start sites of conserved ciliary genes (black line) or all other genes (grey line).
